# Supplementary material for: Screening and treatment to reduce severe hyperbilirubinaemia in infants in primary care (STARSHIP): a factorial stepped-wedge cluster randomised controlled trial protocol
Source: BMJ Open. 2019 Apr 20;9(4):e028270. doi: 10.1136/bmjopen-2018-028270 (PMC6500291; doi:10.1136/bmjopen-2018-028270)
Supplement: Author's manuscript [file bmjopen-2018-028270.draft_revisions.pdf]

BMJ Open is committed to open peer review. As part of this commitment we make the peer review history of every article we publish publicly available.

When an article is published we post the peer reviewers' comments and the authors' responses online. We also post the versions of the paper that were used during peer review. These are the versions that the peer review comments apply to.

The versions of the paper that follow are the versions that were submitted during the peer review process. They are not the versions of record or the final published versions. They should not be cited or distributed as the published version of this manuscript.

BMJ Open is an open access journal and the full, final, typeset and author-corrected version of record of the manuscript is available on our site with no access controls, subscription charges or pay-per-view fees (http://bmjopen.bmj.com).

If you have any questions on BMJ Open's open peer review process please email info.bmjopen@bmj.com

BMJ Open

Screening and TreAtment to Reduce Severe Hyperbilirubinaemia in Infants in Primary care (STARSHIP): a factorial stepped wedge cluster randomised controlled trial protocol

Journal:	*BMJ Open*
Manuscript ID	bmjopen-2018-028270
Article Type:	Protocol
Date Submitted by the Author:	30-Nov-2018

Complete List of Authors:	van der Geest, Berthe; Erasmus MC, Department of Obstetrics and Gynaecology, Division of Obstetrics and Prenatal Medicine; Erasmus MC Sophia, Department of Paediatrics, Division of Neonatology<br>de Graaf, J; Erasmus Medical Center, Department of Obstetrics and Gynaecology, Division of Obstetrics and Prenatal Medicine<br>Bertens, Loes; Erasmus MC, Department of Obstetrics and Gynaecology, Division of Obstetrics and Prenatal Medicine<br>Poley, Marten; Erasmus Universiteit Rotterdam, Medical Technology Assessment (iMTA); Erasmus MC Sophia, Intensive care an department of Pediatric Surgery<br>Ista, Erwin; Erasmus MC Sophia, Department of Paediatrics, Intensive Care Unit; Erasmus MC, Department of Internal Medicine, Nursing Science<br>Kornelisse, René; Erasmus MC Sophia, Department of Paediatrics, Division of Neonatology<br>Reiss, Irwin; Erasmus MC Sophia, Department of Paediatrics, Division of Neonatology<br>Steegers, Eric; Erasmus MC, Department of Obstetrics and Gynaecology, Division of Obstetrics and Prenatal Medicine<br>Been, JV; Erasmus MC, Department of Obstetrics and Gynaecology, Division of Obstetrics and Prenatal Medicine; Erasmus MC Sophia, Department of Paediatrics, Division of Neonatology

Keywords:	NEONATOLOGY, neonatal unconjugated hyperbilirubinemia, neonatal jaundice, transcutaneous bilirubin measurement, phototherapy, PRIMARY CARE

SCHOLARONE™
Manuscripts

Screening and TreAtment to Reduce Severe Hyperbilirubinaemia in Infants in Primary care (STARSHIP): a factorial stepped wedge cluster randomised controlled trial protocol

Berthe A.M. van der Geest[1,2], Johanna P. de Graaf[1], Loes C.M. Bertens[1], Marten J. Poley[3,4], Erwin Ista[5,6], René F. Kornelisse[2], Irwin K.M. Reiss[2], Eric A.P. Steegers[1], Jasper V. Been[1,2,7]*

On behalf of the STARSHIP study group: Martin G.A. Baartmans[8], Jolita Bekhof[9], Harry Buijs[10,11], Jan Erik Bunt[12], Peter H. Dijk[13], Marja C. Huizer[10,11,14], Christian V. Hulzebos[13], Ralph W.J. Leunissen[15], Beata Pazur[14], Ben J.P.W. Snoeren[16], Bente de Vries[17], Leo Wewerinke[18].

*Corresponding author: Jasper V. Been, Division of Neonatology, Department of Paediatrics, Erasmus MC – Sophia Children's Hospital, University Medical Centre, PO Box 2060, 3000 CB Rotterdam, Netherlands;

E: j.been@erasmusmc.nl; T: +31-10-7037048; F: +31-10-7036815.

[1]Department of Obstetrics and Gynaecology, Division of Obstetrics and Prenatal Medicine, Erasmus MC, University Medical Centre Rotterdam, the Netherlands.

[2]Department of Paediatrics, Division of Neonatology, Erasmus MC – Sophia Children's Hospital, University Medical Centre Rotterdam, the Netherlands.

[3]Institute for Medical Technology Assessment (iMTA), Erasmus University Rotterdam, Rotterdam, the Netherlands.

[4]Intensive Care and Department of Paediatric Surgery, Erasmus MC – Sophia Children's Hospital, University Medical Centre Rotterdam, The Netherlands.

For peer review only - http://bmjopen.bmj.com/site/about/guidelines.xhtml

[5]Department of Paediatrics, Intensive Care Unit, Erasmus MC – Sophia Children's Hospital, University Medical Centre Rotterdam, the Netherlands.

[6]Department of Internal Medicine, Nursing Science, Erasmus MC, University Medical Centre Rotterdam, the Netherlands

[7]Department of Public Health, Erasmus MC, University Medical Centre Rotterdam, the Netherlands.

[8]Department of Paediatrics, Maasstad Hospital, Rotterdam, the Netherlands.

[9]Department of Paediatrics, Isala – Amalia Children's Clinic, Zwolle, the Netherlands.

[10]Primary care birth centre Haga, The Hague, the Netherlands.

[11]Primary care birth centre Maasstad, Rotterdam, the Netherlands.

[12]Department of Paediatrics, Elisabeth-TweeSteden Hospital, Tilburg, the Netherlands.

[13]Department of Neonatology, University Medical Centre Groningen – Beatrix Children's Hospital, University of Groningen, Groningen, the Netherlands.

[14]Primary care birth centre Rotterdam Noord, Rotterdam, the Netherlands.

[15]Department of Paediatrics, Haaglanden Medical Centre Westeinde, The Hague, the Netherlands.

[16]Primary care birth centre Fam, Tilburg, the Netherlands.

[17]Primary care birth centre Westeinde, The Hague, the Netherlands.

[18]Department of Paediatrics, Haga Hospital – Juliana Children's Hospital, The Hague, the Netherlands.

Word count: 4037

For peer review only

ABSTRACT

Introduction:

Jaundice, caused by hyperbilirubinaemia, is a physiological phenomenon in the neonatal period. However, severe hyperbilirubinaemia – when left untreated – may cause kernicterus, a severe condition resulting in lifelong neurologic disabilities. Although commonly applied, visual inspection is ineffective in identifying severe hyperbilirubinaemia. We aim to investigate whether, among babies cared for in primary care: 1. transcutaneous bilirubinometry (TcB) screening can help reduce severe hyperbilirubinaemia, and 2. primary care-based (versus hospital-based) phototherapy can help reduce hospital admissions.

Methods and analysis:

A factorial stepped-wedge cluster randomised controlled trial will be conducted in seven Dutch primary care birth centres (PCBC). Neonates born after 35 weeks of gestation, cared for at a participating PCBC during at least two days within the first week of life are eligible, provided they have not received phototherapy before. According to the stepped-wedge design, following a phase of 'usual care' (visual assessment and selective total serum bilirubin (TSB) quantification), either daily TcB measurement or, if indicated, phototherapy in the PCBC will be implemented (phase 2). In phase 3, both interventions will be evaluated in each PCBC. We aim to include 5500 neonates over 3 years.

Primary outcomes are assessed at 14 days of life: 1. the proportion of neonates having experienced severe hyperbilirubinaemia (for the TcB screening intervention), defined as a TSB above the mean of the phototherapy and the exchange transfusion threshold, and 2. the proportion of neonates having required hospital admission for hyperbilirubinaemia treatment (for the phototherapy intervention in primary care).

Ethics and dissemination:

This study has been approved by the Medical Research Ethics Committee of the Erasmus MC Rotterdam, the Netherlands (MEC-2017-473). Written parental informed consent will be obtained.

For peer review only - http://bmjopen.bmj.com/site/about/guidelines.xhtml

Results from this study will be published in peer-reviewed journals and presented at (inter)national meetings.

Trial registration number: NTR7187 (Dutch Trial Registry)

Protocol date and version: 19 June 2018, version 3.1

Keywords: neonatal unconjugated hyperbilirubinemia, neonatal jaundice, kernicterus, transcutaneous bilirubin measurement, phototherapy, primary health care, birth center, randomized controlled trial, stepped wedge, cluster, factorial.

For peer review only

For peer review only - http://bmjopen.bmj.com/site/about/guidelines.xhtml

STRENGHTS AND LIMITATIONS OF THIS STUDY

- This study is the first randomised controlled trial to assess the (cost-)effectiveness of transcutaneous bilirubin measurement as a universal screening tool for potentially severe jaundice, addressing a knowledge gap highlighted in several national guidelines.
- This study is also the first randomised controlled trial to assess the (cost-)effectiveness of phototherapy outside the hospital setting.
- The stepped-wedge design of this study allows all participating primary care birth centres to have introduced both interventions as standard care towards the end of the study, which will aid implementation if (cost-)effectiveness of the interventions is demonstrated.
- A limitation is that parents and care providers cannot be blinded to allocation of the interventions.

For peer review only

For peer review only - http://bmjopen.bmj.com/site/about/guidelines.xhtml

INTRODUCTION

In newborns, severe hyperbilirubinaemia may cause brain damage when it is not recognised or is left untreated.[1,2] This spectrum of neurological sequelae is called kernicterus spectrum disorders (KSD) with acute bilirubin encephalopathy and its consequences at the extreme end and more subtle disorders at the other.[1,3] KSD also encompasses "classical kernicterus", a chronic, irreversible, neuropathological disorder involving neuromotor dysfunction, auditory disorders, and oculomotor impairments.[1-4]

In high income countries an estimated 1 in 67,000 neonates develops classical kernicterus.[5,6]

Timely recognition of potentially severe jaundice, caused by hyperbilirubinaemia, is essential to prevent KSD.[7,8] Traditionally, jaundice is identified via visual inspection by maternity care professionals, midwives or parents. If potentially severe jaundice is suspected, total serum bilirubin (TSB) is quantified in blood taken via a skin prick to assess the need for treatment.[9] However, visual inspection of neonatal jaundice is proven to be inaccurate in detecting hyperbilirubinaemia [10,11] and therefore ineffective in preventing kernicterus.[12]

Guidelines in several high-income countries advise universal screening for jaundice in newborns using TSB or transcutaneous bilirubin (TcB) measurement.[10,13] Several observational studies have shown a good correlation between TSB and TcB measurements in the neonatal period.[14,15] A programmatic evaluation of a TcB-based screening programme in Canada demonstrated a 55% reduction in severe hyperbilirubinaemia and related health care utilisation as compared to visual inspection with selective TSB quantification.[16] Similar findings were obtained in a retrospective multicentre study evaluating a screening program using standard versus selective TSB or TcB measurement in the United States.[17] Universal screening for neonatal jaundice in the primary care setting has the potential to reduce the incidence of severe hyperbilirubinaemia.[2,18] This would also bring down the number of patients who require exchange transfusion, which is a highly invasive treatment that carries significant health risks and substantial costs.

For peer review only - http://bmjopen.bmj.com/site/about/guidelines.xhtml

Hyperbilirubinaemia is usually treated with phototherapy in a hospital setting, which may be applied via various modalities.[2] Fibreoptic phototherapy using a underneath device (i.e. mattress) is effective in reducing TSB levels, is safe,[19] and meets existing guidelines.[9] There is some evidence to suggest that fibreoptic phototherapy may safely and effectively be used in the primary care setting as well.[14,20,21] Institution of phototherapy in the primary care setting may reduce the number of neonates admitted to hospital for hyperbilirubinaemia treatment and reduce associated costs.[22]

Whereas universal TcB screening for neonatal hyperbilirubinaemia and phototherapy provided in primary care have the potential to be effective and cost-effective via reducing the incidence of severe hyperbilirubinaemia and the need for hospitalisation,[14,23] evidence to support these assertions is currently lacking, while in today's health care arena cost-effectiveness considerations are becoming increasingly important.

We hypothesise that non-invasive screening for neonatal hyperbilirubinaemia using TcB and application of phototherapy in primary care will (cost-)effectively reduce the incidence of severe hyperbilirubinaemia and the need for hospital admission for hyperbilirubinaemia treatment. Here, we present our protocol for a factorial stepped-wedge cluster randomised controlled trial (RCT) among newborns cared for in primary care to test these hypotheses.

For peer review only

METHODS AND ANALYSIS

Study design

We will conduct a factorial stepped-wedge cluster RCT in seven primary care birth centres (PCBCs) in the Netherlands to evaluate among newborns the effectiveness of: (1) screening for hyperbilirubinaemia using daily TcB quantification to reduce the incidence of severe hyperbilirubinaemia, and (2) treatment of hyperbilirubinaemia (if indicated) using phototherapy instituted within the PCBC to reduce the need for hospital admission. Accordingly, each PCBC will

For peer review only - http://bmjopen.bmj.com/site/about/guidelines.xhtml

start with (1) a control phase in which usual care is evaluated, followed by (2) a second phase in which one of both interventions is evaluated, followed by (3) a final phase in which both interventions are evaluated in parallel. More detail is provided below.

Dutch perinatal care system

In the Netherlands, a significant proportion of women (30% in 2016) give birth in a primary care setting (i.e. at home or in a PCBC) under supervision of a community midwife.[24] In addition, many who deliver in a hospital setting are discharged home or to a PCBC within 24 hours after delivery. Maternity care assistants provide maternity care when the neonate and the mother are at home or in a PCBC, under supervision of the community midwife (who will visit at least 3 times in the first week).[25] The community midwife will consult the paediatrician of an affiliated nearby hospital if a potential clinical problem such as hyperbilirubinaemia presents in the neonate. Neonates with a gestational age above 35 weeks who have been discharged from the hospital are especially at risk of developing severe unconjugated hyperbilirubinaemia.[26]

Participants

Study sites

This study will be performed in seven PCBCs in the Netherlands. In these PCBCs altogether approximately 5,000 neonates and their mothers are admitted for at least 48 hours each year. Each of the five largest PCBCs will be considered a cluster in the context of our cluster RCT. The two smallest PCBCs will be paired and will form a cluster together. A PCBC was selected for the study if it facilitated provision of maternity care during the first days postpartum (that is, some PCBCs only facilitate deliveries and do not provide care beyond the first 24 hours after delivery).

Patient eligibility

Neonates are eligible for inclusion in the study if:

For peer review only - http://bmjopen.bmj.com/site/about/guidelines.xhtml

- The neonate was born after 35 completed weeks of gestation.

  Note: Only neonates with a gestational age of 37 weeks or higher are born in primary care; Nevertheless, a neonate may be eligible when born in the hospital between 35 and 37 weeks gestational age and then discharged to a PCBC within seven days after birth.
- The neonate is admitted to a participating PCBC within the first week of life.
- The neonate is expected to remain admitted for at least two days (to allow for serial TcB measurements to take place).
- Signed informed consent (IC) is provided by parent(s) or primary caregiver(s).

Neonates are not eligible if:

- The neonate received phototherapy previously or is currently receiving phototherapy (reliability of TcB measurement is reduced in neonates who are receiving or have received phototherapy).[14]
- Parents do not have sufficient understanding of the Dutch language to be able to comprehend the patient information sheet and questionnaire.

The planned enrolment of participants is between July 2018 and July 2021.

Interventions and control

In this study, two interventions will be assessed in a 2x2 factorial design to determine the effectiveness of each intervention separately. Timing of implementation of each intervention will be allocated at the PCBC level.

Control group: usual care

During this initial study phase, usual care will be evaluated. Accordingly, visual inspection is used to identify jaundice, as advised in the Dutch national guideline for neonatal hyperbilirubinaemia.[9] When potentially severe jaundice is suspected, a skin prick will be performed to determine TSB. The TSB level will be plotted on the Dutch nomogram [27] (which is similar to the nomogram of the

For peer review only - http://bmjopen.bmj.com/site/about/guidelines.xhtml

American Academy of Pediatrics and explained in the text box below) and be discussed with the affiliated paediatrician, according to local procedures. If treatment for hyperbilirubinaemia is indicated, as judged by the affiliated paediatrician, the neonate will be admitted to the hospital for this purpose.

Intervention 1: TcB measurement as a non-invasive screening tool

During the study phase in which this intervention is tested, TcB will be measured daily starting from 24 hours of age using the Dräger JM-105, a CE marked, and validated hand-held bilirubinometer, in all included neonates admitted to a PCBC allocated to this intervention.[28-31] If TcB exceeds the phototherapy threshold, as determined by the Dutch TSB nomogram for neonates born after 35 weeks of gestation [9], TSB will be quantified. Because the nomograms were originally designed to be applied to TSB measurements and because TcB measurements have an inaccuracy of maximally 50 μmol/L (i.e. 2.9 mg/dL),[14,32] TSB will also be quantified if TcB is less than 50 μmol/L below the phototherapy treatment threshold. The TSB level obtained will be discussed with an affiliated paediatrician (of the nearby hospital) in order to determine the need for phototherapy, again based on the Dutch nomogram.[9] A second TcB measurement on the same day may be performed at the discretion of the maternity care professional (e.g. if the TcB level is close to the threshold for measuring TSB).

Following institution of phototherapy, bilirubin levels will be monitored using TSB rather than TcB given the reduced reliability of TcB in neonates receiving or having received phototherapy.[14]

For peer review only

The Dutch TSB nomogram [27]

The Dutch TSB nomogram has the same values as the nomogram of the American Academy of Pediatrics. The nomogram consists of three risk categories: lower risk, medium risk and higher risk. The risk assessment is based on gestational age and several risk factors (blood group antagonism, other haemolytic disease, birth asphyxia, suspicion of infection/sepsis, the neonate being ill or drowsy, and serum albumin level below 30 g/L). The need for phototherapy and exchange transfusion depend on the risk assessment and the postnatal age of the neonate. A translated version of the Dutch TSB nomogram is depicted in Figure 1.

Figure 1: phototherapy and exchange transfusion thresholds for neonates with a gestational age of 35 weeks or more (TSB = total serum bilirubin; PT = phototherapy; ET = exchange transfusion; AS = Apgar score). Translated from Dutch. The Dutch nomogram can be downloaded from http://babyzietgeel.nl/kinderarts/hulpmiddelen/diagnostiek/bilicurve35wkn.php.

review only

Intervention 2: phototherapy in the PCBC

During this study phase the default location for application of phototherapy – if clinically indicated – will be the PCBC, rather than the hospital. The indication for phototherapy is made by a paediatrician in the affiliated hospital based on the TSB level, according to the Dutch nomogram.[9] Phototherapy in the PCBC will be provided using the GE Healthcare BiliSoft Large, a commercially available, CE marked and validated phototherapy mattress using blue LED-light.[33] This phototherapy mattress is suitable for application in primary care as well as in the hospital setting.

The affiliated paediatrician will decide whether other blood tests have to be performed to determine the cause of hyperbilirubinaemia and at what time points follow-up TSB measurements need to be taken. Based on the results of these TSB measurements (dis)continuation of phototherapy and further follow-up is determined by the affiliated paediatrician. The affiliated paediatrician may decide to admit a neonate to the hospital for further hyperbilirubinaemia treatment at any time if

For peer review only - http://bmjopen.bmj.com/site/about/guidelines.xhtml

there are strong reasons to do so; these reasons will be recorded. Phototherapy will not be applied in the PCBC in neonates who have hyperbilirubinaemia above the phototherapy threshold within 24 hours after birth, since this is considered to represent a likely pathologic cause of hyperbilirubinaemia and bilirubin levels may rise swiftly in these neonates.[9]

Each PCBC will start with a time period without any intervention (control period), followed by a time period with only one of the interventions, and then a time period with both interventions. The inclusion of neonates will take 2.5 years per PCBC. The sequence and the timing at which the PCBC will switch over to the next intervention period will be determined by random allocation. The allocation scheme with the possible sequences of the interventions is depicted in Figure 2. The time blocks will take 4 months, except for the first and the last block. These two blocks will cover 5.5 months, because, to be able to make a comparison within a cluster, a sufficient number of participants in the control period (C) and in the period with both interventions (I1+I2) should be included, even when a smaller PCBC is randomised to time line 1 or 6.

Figure 2: allocation scheme (C = control; I1 = intervention 1: TcB measurement; I2 = intervention 2: phototherapy in PCBC)

A flowchart with an overview of the study procedures with the different intervention phases and possible orders of intervention phases from the perspective of a participant is displayed in Figure 3.

Figure 3: flowchart for study inclusion

Primary outcomes

For peer review only - http://bmjopen.bmj.com/site/about/guidelines.xhtml

The two interventions each have their own primary outcome. The primary outcome for TcB measurement as a screening tool for jaundice will be the proportion of neonates having severe hyperbilirubinaemia at any time point within the first 14 days of life. Severe hyperbilirubinaemia is defined as a TSB level above the mean of the phototherapy and exchange transfusion threshold, according to the Dutch nomogram; [9] i.e.:

$$TSB > \frac{phototherapy\ threshold + exchange\ transfusion\ threshold}{2}$$

For the second intervention, phototherapy in the PCBC, the primary outcome will be the proportion of neonates requiring hospital admission for hyperbilirubinaemia treatment within the first 14 days of life. Hyperbilirubinaemia treatment will be considered the indication for hospital admission if the neonate received phototherapy or an exchange transfusion for hyperbilirubinaemia within 24 hours after hospital admission.

Secondary outcomes

The secondary outcomes will also be assessed within the first 14 days of life (except for kernicterus) and are listed in Table 1.

Table 1: secondary outcomes

Secondary outcomes		
**Assessed in**	**Outcome**	**Recorded by**
**All neonates**	Number of times TcB quantified	Maternity care professional
	Individual TcB readings	Maternity care professional
	Highest TSB level	Maternity care professional and/or EMR in hospital
	Number of neonates having TSB quantified	Maternity care professional
	Number of times blood taken for TSB quantification	Maternity care professional and/or EMR in hospital
	Individual TSB levels	Maternity care professional and/or EMR

For peer review only - http://bmjopen.bmj.com/site/about/guidelines.xhtml

		in hospital
	Number of times blood taken for TSB quantification before start of phototherapy	Maternity care professional and/or EMR in hospital
	Number of neonates receiving phototherapy	Maternity care professional and/or EMR in hospital
	Duration (hours) of phototherapy	Maternity care professional and/or EMR in hospital
	Number of neonates having a TSB level above exchange transfusion threshold [9]	Maternity care professional and/or EMR in hospital
	Number of neonates who actually received an exchange transfusion	EMR in hospital
	Number of neonates having kernicterus*	EMR in hospital and/or EMR at general practitioner
	Duration of stay in the PCBC	Maternity care professional
	Duration of hospital stay following initial PCBC admission (if relevant)	EMR in hospital
	Number of transfers between PCBCs/hospitals	Maternity care professional and parental questionnaire
	Number of times affiliated paediatrician consulted	Maternity care professional
	Experience of parents regarding hyperbilirubinaemia assessment and treatment	Parental questionnaire
	Experience of attending maternity care personnel regarding the daily practice of hyperbilirubinaemia assessment and treatment during control and intervention periods, including facilitators and barriers for implementation	Maternity care assistant questionnaire
**Within the group included in the intervention period with phototherapy in the PCBC**	The number of neonates in whom phototherapy was initiated in the hospital, including reasons for this hospital admission	EMR in hospital
	The number of neonates requiring subsequent hospital admission for hyperbilirubinaemia treatment (i.e. after initiation of phototherapy in the PCBC), including reasons for this 'treatment failure'	EMR in hospital

EMR = electronic medical record

For peer review only - http://bmjopen.bmj.com/site/about/guidelines.xhtml

*The diagnosis of kernicterus will be made by combining clinical signs and symptoms and additional investigations (e.g. cerebral ultrasound, MRI) up to one year after birth in every neonate who had a TSB level above the exchange transfusion threshold level.

Cost effectiveness analysis

A cost effectiveness analysis (CEA) will be performed from the health care perspective. In the CEA the costs and outcomes of the interventions, compared to 'usual care', will be identified, measured, and valued.

For all of the following items, health care use will be measured: TSB and TcB tests, phototherapy in the hospital, phototherapy in the PCBC, exchange transfusions, PCBC and/or hospital admission days, and ambulance transportation to a hospital. These data will be recorded during the study and/or retrieved from the electronic information systems of the participating PCBCs and affiliated hospitals.

Then, integral unit prices will be calculated using real economic cost prices or using standard cost-prices for health economic evaluations.[34] Unit prices will be multiplied by the quantities for each resource used and summed over the separate types of resource to give a total cost per patient.

Regarding the outcomes, the CEA will look at the number of neonates with severe hyperbilirubinaemia (as defined above) and the number of hospital admissions. Data on costs and outcomes will be measured throughout the 14-day observation period. As the final outcome measure of the CEA, incremental cost effectiveness ratios (ICERs) will calculated, expressed as incremental costs per case of severe hyperbilirubinaemia or hospitalisation avoided. Uncertainty in the estimation of the ICERs will be illustrated through cost-effectiveness planes. Where relevant, sensitivity analyses will be performed to assess the robustness of the analyses to certain assumptions.

Sample size

Two sample size calculations are required since two interventions, each with their own outcome, are evaluated. The interventions are considered to be independent of each other and therefore the

For peer review only - http://bmjopen.bmj.com/site/about/guidelines.xhtml

largest sample size among the two sample size calculations is considered appropriate for the entire study. Sample size calculations are based on detecting superiority rather than non-inferiority.

The estimated incidence of severe hyperbilirubinaemia in term neonates in the Netherlands is 1.2% per year.[9] A previous study in Canada regarding screening for unconjugated hyperbilirubinaemia using transcutaneous bilirubinometry among healthy neonates ≥35 weeks of gestation showed a relative reduction in severe hyperbilirubinaemia of 55% following implementation.[16] We therefore hypothesise that the TcB screening intervention will decrease the incidence of severe hyperbilirubinaemia from 1.2% to 0.5% (i.e. by 0.7% points; a relative reduction of 58%). At a power of 80% and a two-sided alpha of 0.05, 2691 neonates per arm are needed to identify this effect.
The estimated proportion of neonates needing phototherapy is 4%.[9] We anticipate that approximately 50% of the neonates with hyperbilirubinaemia will have an indication to be admitted to the hospital (e.g. feeding difficulties in addition to hyperbilirubinaemia or need for intensive phototherapy). Therefore, we hypothesise that the planned application of phototherapy in the PCBC will decrease the need for hospital admission for hyperbilirubinaemia by 2% points (i.e. 50% relative reduction). At a power of 80% and a two-sided alpha of 0.05, 1136 neonates per arm are needed to identify this effect. While our study is a cluster RCT, given the cross-sectional nature of a stepped-wedge design the effect of intracluster correlation on power is minimal, therefore we did not adjust for intracluster correlation.[35]

Based on the largest sample size of 2691 neonates per arm, 5382 neonates are needed in total. We aim to include 5500 neonates to account for a degree of missing data.

Randomisation and blinding

For peer review only - http://bmjopen.bmj.com/site/about/guidelines.xhtml
For peer review only

Each of the participating PCBCs will be randomised to one of the predefined timelines (as depicted in Figure 2 above) by an online randomisation module (www.randomization.com). The two smallest PCBCs will be paired and randomised to the same timeline (see Figure 2).

Given the nature of the treatment in the intervention groups and the control group, blinding of (parents of) participants and health care personnel is not possible. Analyses will be performed by a researcher who is blinded to the PCBC allocation scheme.

For peer review only

Study procedures

All PCBCs in the Netherlands that provide maternity care to mothers and neonates in the first days after delivery were invited to participate. Eligibility of neonates admitted to the PCBC will be assessed by the maternity care or study personnel based on the inclusion and exclusion criteria. Informed consent of parents will be obtained by the maternity care professional. Research nurses and study personnel will support the maternity care professionals in obtaining informed consent and collecting data.

Depending on the allocation and the moment of admission in the PCBC during the study period, the neonate is included in the control period, in a time period where one of the interventions is implemented, or in a time period where both interventions are implemented. Standard operating procedures (SOPs) for each study phase have been developed.

Maternity care personnel of all PCBCs will be trained in the SOPs and in using the transcutaneous bilirubinometer and the phototherapy mattress to ensure the interventions are applied in a professional standardised manner. For this purpose, an e-learning module has been developed (available from: https://xoteur.12change.eu/play.php?template_id=798); in addition, during the study period three training sessions for maternity care professionals are held at each PCBC prior to the start of each subsequent study phase (i.e. intervention implementation).

For peer review only - http://bmjopen.bmj.com/site/about/guidelines.xhtml

Data collection

Data collection at the PCBC will be performed by maternity care personnel. Together with the training for using the transcutaneous bilirubinometer and the phototherapy mattress, maternity care personnel will be trained in adequate data collection. Data collection is described in the SOPs as well.

After inclusion, a maternity care professional of the PCBC will record baseline characteristics of the mother and the neonate (see Table 2) using a standardised registration form at a secured internet page. All participants will receive an anonymised study number.

The maternity care professionals will record several measurements during PCBC stay. These measurements are noted in Table 3. All data will be recorded in standardised case report forms. The study team, the study monitor, and (inter)national supervisory authorities will have access to the data and the final dataset.

Table 2: baseline characteristics

Baseline characteristics	
**Parental characteristics**	Maternal age
	Maternal ethnicity
	Maternal blood group
	Paternal ethnicity
	Presence of haemolytic disease (other than blood group antagonism) in mother or father
**Characteristics of pregnancy and birth**	Parity
	Maternal atypical red-cell alloantibodies during pregnancy
	Gestational age at birth
	Type of delivery: vaginal delivery, vacuum-assisted vaginal delivery, forceps-assisted vaginal delivery, caesarean delivery without vacuum or forceps extraction (before or during delivery), caesarean delivery with vacuum or forceps extraction (before or during delivery).
**Neonatal characteristics**	Date of birth
	Time of birth

For peer review only - http://bmjopen.bmj.com/site/about/guidelines.xhtml

	Sex
	Birth weight (in grams)
	Presence of birth trauma
	Type of feeding
	Neonatal blood group (if known)
	Foetal or neonatal Rhesus factor (if known)
	Direct antiglobulin (Coombs) test (if known)
	Presence of haemolytic disease (other than blood group antagonism)
	Presence of birth asphyxia: Apgar score <5 at 5 minutes or pH <7.0 in umbilical cord blood
	Suspicion of sepsis
	Neonate being ill or drowsy
**Other characteristics**	Siblings who experienced neonatal hyperbilirubinaemia (and cause of hyperbilirubinaemia, if known)

Table 3: daily measurements in PCBC

**Daily measurements**	
Control period	Skin colour: pink, slightly yellow, moderately yellow, quite yellow, very yellow
	Weight (in grams)
	Risk factors for hyperbilirubinaemia (if present)
	TSB values with date and time of measurement (if relevant)
	Decisions made based on TSB (if relevant)
Period with TcB screening (extra measurements in addition to control period)	TcB values measured at forehead and sternum together with date and time of measurement (once a day or two times if indicated)
Phototherapy in PCBC (extra measurements in addition to control period)	Start and end date and time of phototherapy
	Decisions made by the affiliated paediatrician regarding phototherapy

A questionnaire for the parents/caregivers to evaluate their experience will be sent by e-mail 14 days after birth of the neonate. This questionnaire will assess parental satisfaction, burdensomeness of interventions for the neonate, and experienced competence of maternity care professionals. If parents do not have access to the internet or do not respond to the questionnaire by e-mail, they will be approached by regular mail or by phone. The community midwife and/or the general practitioner will be approached if parents do not respond at all. Both after the control and after each intervention

For peer review only - http://bmjopen.bmj.com/site/about/guidelines.xhtml

For peer review only

period, maternity care personnel will be invited to complete a questionnaire about their experiences regarding the daily practice of hyperbilirubinaemia assessment and treatment during these periods (e.g. facilitators and barriers for implementation, sense of competence regarding applying the new interventions, integrating the new interventions in routine care, and the content of the training regarding the new interventions).

Statistical analysis

We will use cluster specific methods because randomisation will be performed at the PCBC level rather than at the individual level. For both primary objectives an intention-to-treat analysis will be performed.

To evaluate whether the occurrence of the primary outcomes of both interventions are different between the study periods with and without the intervention(s), generalised linear mixed models (GLMMs) will be used. GLMMs account for the systematically different observation periods and for clustering in the data.[36] In case of missing data, multiple imputation using chained equations will be used. Relative risks, risk differences, and relative risk reduction with corresponding 95% confidence intervals will be calculated from the GLMMs.

Subgroup analyses to evaluate the effect of the interventions in neonates of different ethnicities (Caucasian and non-Caucasian) and different gestational age groups (<38 weeks of gestation and ≥38 weeks of gestation) will be performed.

We will perform a per-protocol analysis to assess the impact of potential contamination during the first days of a new intervention period as a sensitivity analysis in addition to the intention-to-treat analysis.

Safety

For peer review only

For peer review only - http://bmjopen.bmj.com/site/about/guidelines.xhtml

Adverse events noticed by parents or maternity care professionals will be recorded in the case report forms by the maternity care assistant or study personnel. Serious adverse events will be communicated to the study team within four days. The study team will investigate this event and report it to the sponsor and the Medical Research Ethics Committee of the Erasmus MC Rotterdam. Insurance has been taken out for every participant in this study. The insurance covers any damage caused by the study.

A Data Safety Monitoring Board will not be established, given that the risks of this study are considered negligible. Monitoring of the study will be carried out according to the Guideline for Good Clinical Practice by an independent, qualified monitor at least once a year per PCBC.[37]

Patient and public involvement

The study protocol, study procedures and patient information form were discussed with the regional patient involvement board of the Rijnmond Obstetric District Platform ('District Verloskundig Platform'; DVP) and patient representatives of the Child & Hospital foundation ('Stichting Kind & Ziekenhuis': https://www.kindenziekenhuis.nl/). They consider the project highly relevant from a patient perspective and have agreed to be involved throughout execution, analysis and interpretation, dissemination to patients, and implementation. The involved patient panels have considered the burden of the interventions negligible and underline the potential benefits of the study.

ETHICS AND DISSEMINATION

This study has been approved by the Medical Research Ethics Committee of the Erasmus MC Rotterdam (MEC-2017-473). Collected data will be coded. Only investigators will have access to identification list. Study documents and data will be stored for 15 years after completion of the study. Written informed consent is provided by parents of neonates included in the study. The study is registered in the Dutch Trial Registry (NTR7187).

For peer review only - http://bmjopen.bmj.com/site/about/guidelines.xhtml

The full protocol will be shared at request. Results of this study including the statistical code will be published in peer-reviewed scientific journals and be presented at (inter)national meetings. We intend to provide an anonymised version of the dataset at request. Authorship will be determined according to the guidelines of the International Committee of Medical Journal Editors (ICMJE).[38] No professional writers will be used.

DISCUSSION

Identification and (referral for) treatment of neonatal jaundice is daily practice for maternity care professionals. The continuing occurrence and devastating consequences of KSD underline the urgent need for more effective identification and treatment of at-risk neonates.[1,7] This pragmatic study aims to evaluate the (cost-)effectiveness of (1) TcB measurement as a screening tool for jaundice in a primary care setting and (2) application of phototherapy in the PCBCs in the Netherlands. To the best of our knowledge, this RCT will be the first to assess the (cost-)effectiveness of these diagnostic and treatment modalities.

The study design allows all participating PCBCs to implement both interventions during the study and as such all PCBCs will have implemented the interventions as standard care toward the end of the study. This process will provide valuable information about the facilitators and barriers for implementation that will help upscaling of the interventions if (cost-)effectiveness is confirmed.

A limitation of this study is that parents and care providers cannot practically be blinded to allocation of the interventions. As a result, maternity care personnel or paediatricians may act differently in control and intervention periods. We will try to identify this via the questionnaires addressed at the maternity care assistants and the recording of the underlying reasons for paediatricians to divert from the new practice of applying phototherapy in the PCBC, if relevant. We will furthermore record

For peer review only

the number of times TSB is quantified to evaluate whether this may change following implementation of the screening programme.

For this study we take advantage of the fact that neonates receive primary care within PCBCs rather than at home. This centralised primary care provision is efficient in terms of feasibility of training the maternity care professionals and of minimising costs of transcutaneous bilirubinometers and phototherapy mattresses (i.e. groups of neonates can be covered with individual devices). Moreover, although the Dutch perinatal health care system is quite unique, care provision in PCBCs in the Netherlands is comparable to regular maternity care in many other high-income countries, which promotes generalisability.

We expect the interventions to be similarly effective in the home setting as in the PCBC setting, since the population in the PCBCs in the Netherlands does not differ from that the home setting. The costs in the PCBC setting may however differ from the home setting. Generalisability of the results to other countries and the home setting depends on several aspects, such as characteristics of the population, organisation of the maternity care system, presence of an effective screening program for neonatal hyperbilirubinaemia, and proximity and availability of sufficient health care.

In conclusion, our study will generate useful data about the (cost-)effectiveness of TcB measurement as a universal screening tool for jaundice, potentially reducing the incidence of severe neonatal hyperbilirubinaemia. Several guidelines have highlighted the lack of research into the prevention of severe hyperbilirubinaemia.[9,10,13,39] Although most of these guidelines do not specify where phototherapy should be instituted, it is standard practice in most high-income countries – including the Netherlands – to do so in the hospital setting.[9,10,39] As such, institution of phototherapy in the primary care setting has significant potential to reduce health care costs associated with hospital admission. Furthermore, it promotes mother-child bonding by allowing mother and child to remain together in the same location.

For peer review only

For peer review only - http://bmjopen.bmj.com/site/about/guidelines.xhtml

ACKNOWLEDGEMENTS

We acknowledge The Netherlands Organisation for Health Research and Development and Erasmus MC for their financial support to this research. We thank Rogier de Jonge for his support concerning the design and funding of this study, Verena Werdmüller von Elgg for her support regarding Medical Research Ethics Committee approval, Liesbeth Dusink and Roel Faber for their support regarding preparation for data collection and composing case report forms, Marc Sylva for help with literature review, Nina Witt, Rik Bouman and Jetty van Ginkel for their support regarding legal issues and contact with manufacturers, Imke Theeuwen for support during the training sessions, and other practical issues in the phase before the start of the study. We thank the patient involvement board of the Rijnmond Obstetric District Platform and the patient representatives of the Child & Hospital Foundation for their feedback regarding the patient perspective. We are grateful to the participating PCBCs, maternity care professionals, midwives, and affiliated paediatricians for taking part in this study.

AUTHORS' CONTRIBUTIONS

BAMG and JVB wrote the first version of the manuscript. JVB, JPG, RFK, IKMR and EAPS designed the study and secured funding. JVB and LCMB developed the methodological part of this study. MJP designed the cost-effectiveness analysis. EI designed evaluation of implementation of both interventions. All authors critically revised the manuscript and gave final approval of the version to be published.

FUNDING

This work is supported by a Health Care Efficiency Research grant of The Netherlands Organisation for Health Research and Development (ZonMw), grant number 843002805, and an Erasmus MC Efficiency Research grant, grant number 2016-16107. The study funders were not involved in the

For peer review only - http://bmjopen.bmj.com/site/about/guidelines.xhtml

collection, management, analysis and interpretation of data; writing of the report; or the decision to submit the report for publication.

COMPETING INTERESTS

All authors declare that they do not have any competing interests.

For peer review only

For peer review only - http://bmjopen.bmj.com/site/about/guidelines.xhtml

REFERENCES

1. Shapiro SM. Definition of the clinical spectrum of kernicterus and bilirubin-induced neurologic dysfunction (BIND). *J Perinatol*. 2005 Jan;25(1):54-9.
2. Olusanya BO, Kaplan M, Hansen TWR. Neonatal hyperbilirubinaemia: a global perspective. *The Lancet Child & Adolescent Health*. 2018;2(8):610-20.
3. Bhutani VK, Johnson-Hamerman L. The clinical syndrome of bilirubin-induced neurologic dysfunction. *Semin Fetal Neonatal Med*. 2015 Feb;20(1):6-13.
4. Le Pichon JB, Riordan SM, Watchko J, et al. The Neurological Sequelae of Neonatal Hyperbilirubinemia: Definitions, Diagnosis and Treatment of the Kernicterus Spectrum Disorders (KSDs). *Curr Pediatr Rev*. 2017;13(3):199-209.
5. Slusher TM, Zamora TG, Appiah D, et al. Burden of severe neonatal jaundice: a systematic review and meta-analysis. *BMJ Paediatr Open*. 2017;1(1):e000105.
6. Sgro M, Campbell DM, Kandasamy S, et al. Incidence of chronic bilirubin encephalopathy in Canada, 2007-2008. *Pediatrics*. 2012 Oct;130(4):e886-90.
7. Bousema S, Govaert P, Dudink J, et al. [Kernicterus is preventable but still occurs] Kernicterus is vermijdbaar, maar komt nog steeds voor. *Ned Tijdschr Geneeskd*. 2015;159:A8518.
8. Sgro M, Campbell D, Shah V. Incidence and causes of severe neonatal hyperbilirubinemia in Canada. *CMAJ*. 2006 Sep 12;175(6):587-90.
9. Nederlandse Vereniging voor Kindergeneeskunde, Kwaliteitsinstituut voor de Gezondheidszorg CBO. [Guideline prevention, diagnosis and treatment of hyperbilirubinaemia among newborns born at a gestational age of more than 35 weeks] Richtlijn preventie, diagnostiek en behandeling van hyperbilirubinemie bij de pasgeborene, geboren na een zwangerschapsduur van meer dan 35 weken. 2008; Available from: https://www.nvk.nl/Portals/0/richtlijnen/hyperbili/richtlijnhyperbili.pdf.
10. Barrington KJ, Sankaran K, Canadian Paediatric Society, Fetus and Newborn Committee. Guidelines for detection, management and prevention of hyperbilirubinemia in term and late preterm newborn infants. *Paediatr Child Health*. 2007;12(Suppl B):1B-12B.
11. Maisels MJ, Clune S, Coleman K, et al. The natural history of jaundice in predominantly breastfed infants. *Pediatrics*. 2014 Aug;134(2):e340-5.
12. Lease M, Whalen B. Assessing jaundice in infants of 35-week gestation and greater. *Curr Opin Pediatr*. 2010 Jun;22(3):352-65.
13. American Academy of Pediatrics, Subcommittee on Hyperbilirubinemia. Management of Hyperbilirubinemia in the Newborn Infant 35 or More Weeks of Gestation. *Pediatrics*. 2004;114:297-316.
14. Bhardwaj K, Locke T, Biringer A, et al. Newborn bilirubin screening for preventing severe hyperbilirubinemia and bilirubin encephalopathy: a rapid review. *Curr Pediatr Rev*. 2017 Jan 10;13(1):67-90.
15. Engle WD, Jackson GL, Engle NG. Transcutaneous bilirubinometry. *Semin Perinatol*. 2014 Nov;38(7):438-51.
16. Wainer S, Parmar SM, Allegro D, et al. Impact of a transcutaneous bilirubinometry program on resource utilization and severe hyperbilirubinemia. *Pediatrics*. 2012 Jan;129(1):77-86.
17. Eggert LD, Wiedmeier SE, Wilson J, et al. The effect of instituting a prehospital-discharge newborn bilirubin screening program in an 18-hospital health system. *Pediatrics*. 2006 May;117(5):e855-62.
18. Bhutani VK, Johnson LH, Jeffrey Maisels M, et al. Kernicterus: epidemiological strategies for its prevention through systems-based approaches. *J Perinatol*. 2004 Oct;24(10):650-62.
19. Kumar P, Chawla D, Deorari A. Light-emitting diode phototherapy for unconjugated hyperbilirubinaemia in neonates. *Cochrane Database Syst Rev*. 2011 Dec 07(12):CD007969.
20. Yilmaz A, Ozkiraz S, Akcan AB, et al. Low-cost home-use light-emitting-diode phototherapy as an alternative to conventional methods. *J Trop Pediatr*. 2015 Apr;61(2):113-8.
21. Walls M, Wright A, Fowlie P, et al. Home phototherapy in the United Kingdom. *Arch Dis Child Fetal Neonatal Ed*. 2004 May;89(3):F282.

For peer review only - http://bmjopen.bmj.com/site/about/guidelines.xhtml

22. Battersby C, Michaelides S, Upton M, et al. Term admissions to neonatal units in England: a role for transitional care? A retrospective cohort study. *BMJ Open*. 2017 May 29;7(5):e016050.
23. McClean S, Baerg K, Smith-Fehr J, et al. Cost savings with transcutaneous screening versus total serum bilirubin measurement for newborn jaundice in hospital and community settings: a cost-minimization analysis. *CMAJ Open*. 2018 Jul-Sep;6(3):E285-E91.
24. Perined. [Perinatal Care in the Netherlands 2016] Perinatale Zorg in Nederland 2016. Utrecht: Perined, 2018.
25. de Boer J, Zondag L. [Multidisciplinary guideline postnatal care] Multidisciplinaire richtlijn Postnatale Zorg - Verloskundige basiszorg voor moeder en kind. Koninklijke Nederlandse Organisatie van Verloskundigen; 2018; Available from: https://www.knov.nl/serve/file/knov.nl/knov_downloads/2882/file/Postnatale_zorg_opgemaakte_versie_door_IB_md_10_aug_2018.pdf.
26. Gotink MJ, Benders MJ, Lavrijsen SW, et al. Severe neonatal hyperbilirubinemia in the Netherlands. *Neonatology*. 2013;104(2):137-42.
27. Dijk PH, de Vries TW, de Beer JJ. [Guideline 'Prevention, diagnosis and treatment of hyperbilirubinemia in the neonate with a gestational age of 35 or more weeks'] Richtlijn 'Preventie, diagnostiek en behandeling bij de pasgeborene, geboren na een zwangerschapsduur van meer dan 35 weken'. *Ned Tijdschr Geneeskd*. 2009;153(A93).
28. Raimondi F, Lama S, Landolfo F, et al. Measuring transcutaneous bilirubin: a comparative analysis of three devices on a multiracial population. *BMC Pediatr*. 2012 Jun 14;12:70.
29. Nagar G, Vandermeer B, Campbell S, et al. Reliability of transcutaneous bilirubin devices in preterm infants: a systematic review. *Pediatrics*. 2013 Nov;132(5):871-81.
30. Jones DF, McRea AR, Knowles JD, et al. A Prospective Comparison of Transcutaneous and Serum Bilirubin Within Brief Time Intervals. *Clin Pediatr (Phila)*. 2017 Oct;56(11):1013-7.
31. Romagnoli C, Zecca E, Catenazzi P, et al. Transcutaneous bilirubin measurement: comparison of Respironics BiliCheck and JM-103 in a normal newborn population. *Clin Biochem*. 2012 Jun;45(9):659-62.
32. Maisels MJ. Transcutaneous bilirubinometry. *Neoreviews*. 2006;7(5):e217-e25.
33. Bhutani VK. Phototherapy to prevent severe neonatal hyperbilirubinemia in the newborn infant 35 or more weeks of gestation. *Pediatrics*. 2011:e1046-52.
34. Hakkaart-van Roijen L, van der Linden N, Bouwmans C, et al. [Costing manual: Methodology of costing research and reference prices for economic evaluation in health care] Kostenhandleiding: Methodologie van kostenonderzoek en referentieprijzen voor economische evaluaties in de gezondheidszorg. 2015.
35. de Hoop E, van der Tweel I, van der Graaf R, et al. The need to balance merits and limitations from different disciplines when considering the stepped wedge cluster randomized trial design. *BMC Med Res Methodol*. 2015 Oct 30;15:93.
36. Hemming K, Haines TP, Chilton PJ, et al. The stepped wedge cluster randomised trial: rationale, design, analysis, and reporting. *BMJ : British Medical Journal*. 2015;350.
37. International Council for Harmonisation of Technical Requirements for Pharmaceuticals for Human Use (ICH). Guideline for Good Clinical Practice ICH. 2016.
38. International Committee of Medical Journal Editors. Defining the Role of Authors and Contributors. 2017 [cited 2018 November 22]; Available from: http://www.icmje.org/recommendations/browse/roles-and-responsibilities/defining-the-role-of-authors-and-contributors.html.
39. National Collaborating Centre for Women's and Children's Health. Neonatal jaundice. National Institute for Health and Clinical Excellence; 2010; Available from: https://www.ncbi.nlm.nih.gov/pubmedhealth/PMH0016565/pdf/PubMedHealth_PMH0016565.pdf.

For peer review only - http://bmjopen.bmj.com/site/about/guidelines.xhtml

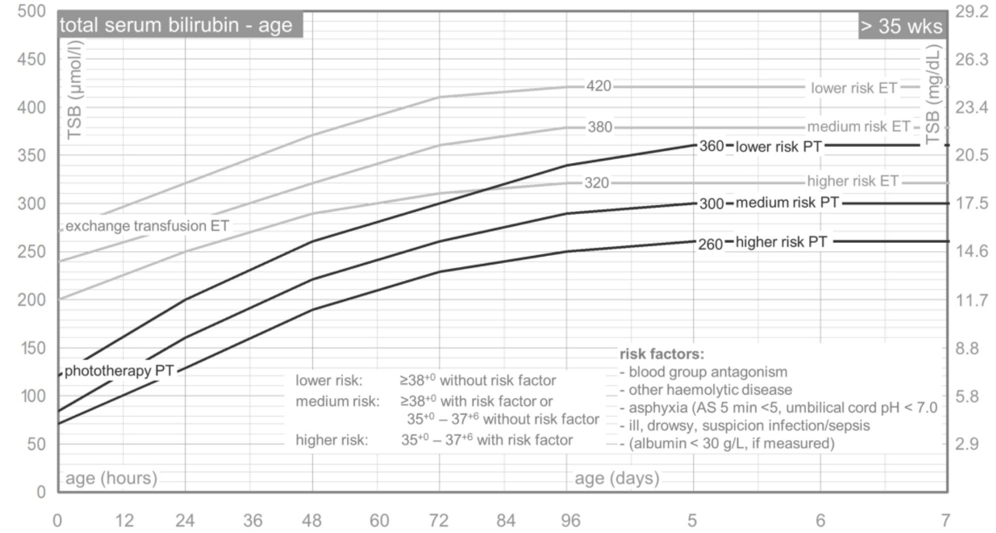

Figure 1: phototherapy and exchange transfusion thresholds for neonates with a gestational age of 35 weeks or more (TSB = total serum bilirubin; PT = phototherapy; ET = exchange transfusion; AS = Apgar score). Translated from Dutch.
The Dutch nomogram can be downloaded from http://babyzietgeel.nl/kinderarts/hulpmiddelen/diagnostiek/bilicurve35wkn.php.

173x99mm (300 x 300 DPI)

Cluster	Time →						
1	C	I1	I1	I1	I1	I1	I1 + I2
2	C	C	I1	I1	I1	I1 + I2	I1 + I2
3	C	C	C	I1	I1 + I2	I1 + I2	I1 + I2
4	C	C	C	I2	I1 + I2	I1 + I2	I1 + I2
5	C	C	I2	I2	I2	I1 + I2	I1 + I2
6	C	I2	I2	I2	I2	I2	I1 + I2

Figure 2: allocation scheme (C = control; I1 = intervention 1: TcB measurement; I2 = intervention 2: phototherapy in PCBC)

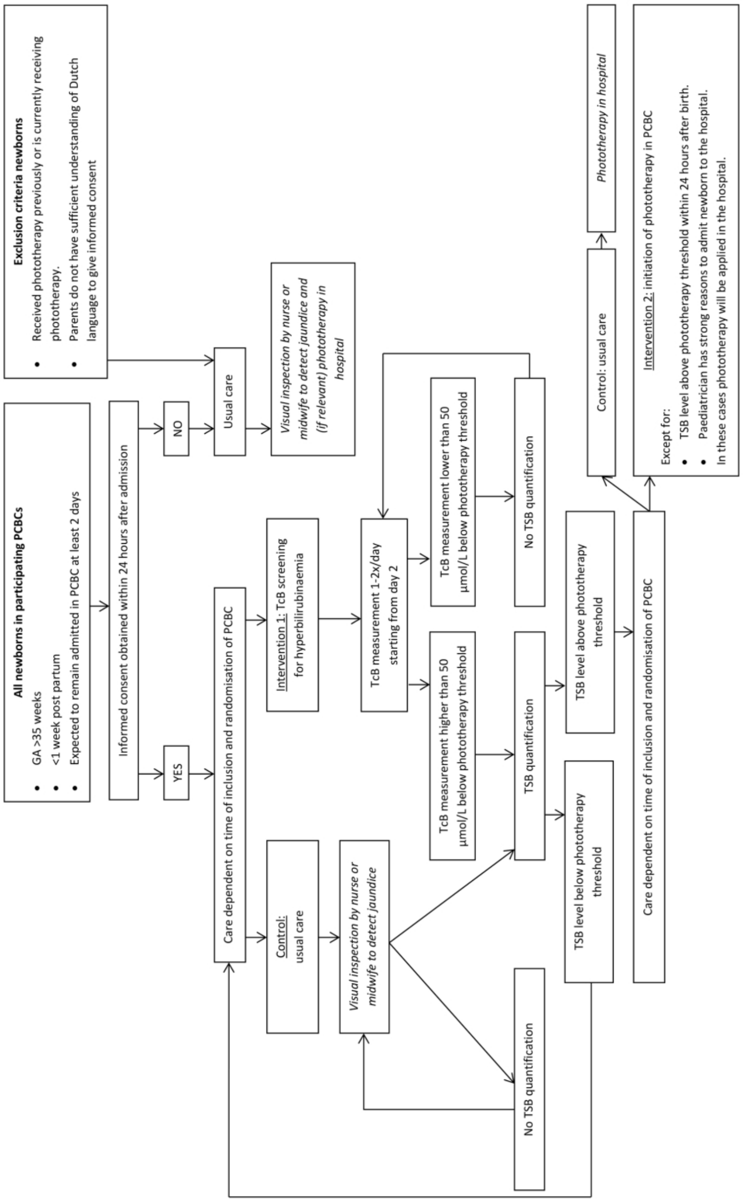

Figure 3: flowchart for study inclusion

146x233mm (300 x 300 DPI)

STANDARD PROTOCOL ITEMS: RECOMMENDATIONS FOR INTERVENTIONAL TRIALS

SPIRIT 2013 Checklist: Recommended items to address in a clinical trial protocol and related documents*

Section/item	Item No	Description
**Administrative information**		
Title	1	Descriptive title identifying the study design, population, interventions, and, if applicable, trial acronym **see page 1**
Trial registration	2a	Trial identifier and registry name. If not yet registered, name of intended registry **see page 4 'Trial registration number' and page 23 'Ethics and dissemination'**
	2b	All items from the World Health Organization Trial Registration Data Set **see http://apps.who.int/trialsearch/Trial2.aspx?TrialID=NTR7187**
Protocol version	3	Date and version identifier **see page 4 'Protocol version and date'**
Funding	4	Sources and types of financial, material, and other support **see page 25 'Funding'**
Roles and responsibilities	5a	Names, affiliations, and roles of protocol contributors **see page 1 and page 25 'Authors' contributions'**
	5b	Name and contact information for the trial sponsor **see page 1 'Corresponding author'**
	5c	Role of study sponsor and funders, if any, in study design; collection, management, analysis, and interpretation of data; writing of the report; and the decision to submit the report for publication, including whether they will have ultimate authority over any of these activities **see page 25 'Funding'**
	5d	Composition, roles, and responsibilities of the coordinating centre, steering committee, endpoint adjudication committee, data management team, and other individuals or groups overseeing the trial, if applicable (see Item 21a for data monitoring committee) **n/a**
**Introduction**		
Background and rationale	6a	Description of research question and justification for undertaking the trial, including summary of relevant studies (published and unpublished) examining benefits and harms for each intervention **see page 6-7 'Introduction'**

For peer review only

	6b	Explanation for choice of comparators **see page 6-7 'Introduction'**
Objectives	7	Specific objectives or hypotheses **see page 7 'Introduction'**
Trial design	8	Description of trial design including type of trial (eg, parallel group, crossover, factorial, single group), allocation ratio, and framework (eg, superiority, equivalence, noninferiority, exploratory) **see page 7-8 'Study design'**

Methods: Participants, interventions, and outcomes

Study setting	9	Description of study settings (eg, community clinic, academic hospital) and list of countries where data will be collected. Reference to where list of study sites can be obtained **see page 8 'Dutch perinatal care system' and 'participants'**
Eligibility criteria	10	Inclusion and exclusion criteria for participants. If applicable, eligibility criteria for study centres and individuals who will perform the interventions (eg, surgeons, psychotherapists) **see page 9 'Patient eligibility'**
Interventions	11a	Interventions for each group with sufficient detail to allow replication, including how and when they will be administered **see page 9-14 'Methods'**
	11b	Criteria for discontinuing or modifying allocated interventions for a given trial participant (eg, drug dose change in response to harms, participant request, or improving/worsening disease) **see page 12 'Intervention 2: phototherapy in the PCBC'**
	11c	Strategies to improve adherence to intervention protocols, and any procedures for monitoring adherence (eg, drug tablet return, laboratory tests) **see page 19 'Study procedures'**
	11d	Relevant concomitant care and interventions that are permitted or prohibited during the trial **n/a**
Outcomes	12	Primary, secondary, and other outcomes, including the specific measurement variable (eg, systolic blood pressure), analysis metric (eg, change from baseline, final value, time to event), method of aggregation (eg, median, proportion), and time point for each outcome. Explanation of the clinical relevance of chosen efficacy and harm outcomes is strongly recommended **see page 15-17 and page 22 'Methods'**
Participant timeline	13	Time schedule of enrolment, interventions (including any run-ins and washouts), assessments, and visits for participants. A schematic diagram is highly recommended (see Figure) **see page 12-14 'Methods'**

For peer review only - http://bmjopen.bmj.com/site/about/guidelines.xhtml

Sample size	14	Estimated number of participants needed to achieve study objectives and how it was determined, including clinical and statistical assumptions supporting any sample size calculations **see page 17-18 'Sample size'**
Recruitment	15	Strategies for achieving adequate participant enrolment to reach target sample size **see page 19 'Study procedures'**

Methods: Assignment of interventions (for controlled trials)

Allocation:

Sequence generation	16a	Method of generating the allocation sequence (eg, computer-generated random numbers), and list of any factors for stratification. To reduce predictability of a random sequence, details of any planned restriction (eg, blocking) should be provided in a separate document that is unavailable to those who enrol participants or assign interventions **see page 18-19 'Randomisation and blinding'**
Allocation concealment mechanism	16b	Mechanism of implementing the allocation sequence (eg, central telephone; sequentially numbered, opaque, sealed envelopes), describing any steps to conceal the sequence until interventions are assigned **n/a**
Implementation	16c	Who will generate the allocation sequence, who will enrol participants, and who will assign participants to interventions **see page 19 'Study procedures' (where applicable)**
Blinding (masking)	17a	Who will be blinded after assignment to interventions (eg, trial participants, care providers, outcome assessors, data analysts), and how **see page 18-19 'Randomisation and blinding'**
	17b	If blinded, circumstances under which unblinding is permissible, and procedure for revealing a participant's allocated intervention during the trial **n/a**

Methods: Data collection, management, and analysis

Data collection methods	18a	Plans for assessment and collection of outcome, baseline, and other trial data, including any related processes to promote data quality (eg, duplicate measurements, training of assessors) and a description of study instruments (eg, questionnaires, laboratory tests) along with their reliability and validity, if known. Reference to where data collection forms can be found, if not in the protocol **see page 19-22 'Study procedures' and 'Data collection'**
	18b	Plans to promote participant retention and complete follow-up, including list of any outcome data to be collected for participants who discontinue or deviate from intervention protocols **see page 21 (where applicable)**

For peer review only - http://bmjopen.bmj.com/site/about/guidelines.xhtml

Data management	19	Plans for data entry, coding, security, and storage, including any related processes to promote data quality (eg, double data entry; range checks for data values). Reference to where details of data management procedures can be found, if not in the protocol **see page 20 (where applicable)**
Statistical methods	20a	Statistical methods for analysing primary and secondary outcomes. Reference to where other details of the statistical analysis plan can be found, if not in the protocol **see page 22 'Statistical analysis'**
	20b	Methods for any additional analyses (eg, subgroup and adjusted analyses) **see page 22 'Statistical analysis'**
	20c	Definition of analysis population relating to protocol non-adherence (eg, as randomised analysis), and any statistical methods to handle missing data (eg, multiple imputation) **see page 22 'Statistical analysis'**
**Methods: Monitoring**		
Data monitoring	21a	Composition of data monitoring committee (DMC); summary of its role and reporting structure; statement of whether it is independent from the sponsor and competing interests; and reference to where further details about its charter can be found, if not in the protocol. Alternatively, an explanation of why a DMC is not needed **see page 22-23 'Safety'**
	21b	Description of any interim analyses and stopping guidelines, including who will have access to these interim results and make the final decision to terminate the trial **n/a**
Harms	22	Plans for collecting, assessing, reporting, and managing solicited and spontaneously reported adverse events and other unintended effects of trial interventions or trial conduct **see page 22-23 'Safety'**
Auditing	23	Frequency and procedures for auditing trial conduct, if any, and whether the process will be independent from investigators and the sponsor **see page 22-23 'Safety'**
**Ethics and dissemination**		
Research ethics approval	24	Plans for seeking research ethics committee/institutional review board (REC/IRB) approval **see page 23 'Ethics and dissemination'**
Protocol amendments	25	Plans for communicating important protocol modifications (eg, changes to eligibility criteria, outcomes, analyses) to relevant parties (eg, investigators, REC/IRBs, trial participants, trial registries, journals, regulators) **n/a**
Consent or assent	26a	Who will obtain informed consent or assent from potential trial participants or authorised surrogates, and how (see Item 32) **see page 19 'Study procedures'**

	26b	Additional consent provisions for collection and use of participant data and biological specimens in ancillary studies, if applicable **n/a**
Confidentiality	27	How personal information about potential and enrolled participants will be collected, shared, and maintained in order to protect confidentiality before, during, and after the trial **see page 20 'Data collection'**
Declaration of interests	28	Financial and other competing interests for principal investigators for the overall trial and each study site **see page 26 'Competing interests'**
Access to data	29	Statement of who will have access to the final trial dataset, and disclosure of contractual agreements that limit such access for investigators **see page 20 'Data collection'**
Ancillary and post-trial care	30	Provisions, if any, for ancillary and post-trial care, and for compensation to those who suffer harm from trial participation **See page 22-23 'Safety'**
Dissemination policy	31a	Plans for investigators and sponsor to communicate trial results to participants, healthcare professionals, the public, and other relevant groups (eg, via publication, reporting in results databases, or other data sharing arrangements), including any publication restrictions **see page 23 'Ethics and dissemination'**
	31b	Authorship eligibility guidelines and any intended use of professional writers **see page 23 'Ethics and dissemination'**
	31c	Plans, if any, for granting public access to the full protocol, participant-level dataset, and statistical code **see page 23 'Ethics and dissemination'**
**Appendices**		
Informed consent materials	32	Model consent form and other related documentation given to participants and authorised surrogates **Patient information form including model consent form are provided as Appendices for the editors.**
Biological specimens	33	Plans for collection, laboratory evaluation, and storage of biological specimens for genetic or molecular analysis in the current trial and for future use in ancillary studies, if applicable **n/a**

*It is strongly recommended that this checklist be read in conjunction with the SPIRIT 2013 Explanation & Elaboration for important clarification on the items. Amendments to the protocol should be tracked and dated. The SPIRIT checklist is copyrighted by the SPIRIT Group under the Creative Commons "Attribution-NonCommercial-NoDerivs 3.0 Unported" license.

For peer review only - http://bmjopen.bmj.com/site/about/guidelines.xhtml

BMJ Open

Screening and TreAtment to Reduce Severe Hyperbilirubinaemia in Infants in Primary care (STARSHIP): a factorial stepped wedge cluster randomised controlled trial protocol

Journal:	*BMJ Open*
Manuscript ID	bmjopen-2018-028270.R1
Article Type:	Protocol
Date Submitted by the Author:	13-Feb-2019
Complete List of Authors:	van der Geest, Berthe; Erasmus MC, Department of Obstetrics and Gynaecology, Division of Obstetrics and Prenatal Medicine; Erasmus MC Sophia, Department of Paediatrics, Division of Neonatology<br>de Graaf, J; Erasmus Medical Center, Department of Obstetrics and Gynaecology, Division of Obstetrics and Prenatal Medicine<br>Bertens, Loes; Erasmus MC, Department of Obstetrics and Gynaecology, Division of Obstetrics and Prenatal Medicine<br>Poley, Marten; Erasmus Universiteit Rotterdam, Medical Technology Assessment (iMTA); Erasmus MC Sophia, Intensive care and department of Pediatric Surgery<br>Ista, Erwin; Erasmus MC Sophia, Department of Paediatrics, Intensive Care Unit; Erasmus MC, Department of Internal Medicine, Nursing Science<br>Kornelisse, René; Erasmus MC Sophia, Department of Paediatrics, Division of Neonatology<br>Reiss, Irwin; Erasmus MC Sophia, Department of Paediatrics, Division of Neonatology<br>Steegers, Eric; Erasmus MC, Department of Obstetrics and Gynaecology, Division of Obstetrics and Prenatal Medicine<br>Been, JV; Erasmus MC, Department of Obstetrics and Gynaecology, Division of Obstetrics and Prenatal Medicine; Erasmus MC Sophia, Department of Paediatrics, Division of Neonatology<br>STARSHIP study group, STARSHIP study group
**Primary Subject Heading**:	Paediatrics
Secondary Subject Heading:	Obstetrics and gynaecology, Public health
Keywords:	NEONATOLOGY, neonatal unconjugated hyperbilirubinemia, neonatal jaundice, transcutaneous bilirubin measurement, phototherapy, PRIMARY CARE

Screening and TreAtment to Reduce Severe Hyperbilirubinaemia in Infants in Primary care (STARSHIP): a factorial stepped wedge cluster randomised controlled trial protocol

Berthe A.M. van der Geest[1,2], Johanna P. de Graaf[1], Loes C.M. Bertens[1], Marten J. Poley[3,4], Erwin Ista[5,6], René F. Kornelisse[2], Irwin K.M. Reiss[2], Eric A.P. Steegers[1], Jasper V. Been[1,2,7]*

STARSHIP study group: Martin G.A. Baartmans[8], Jolita Bekhof[9], Harry Buijs[10,11], Jan Erik Bunt[12], Peter H. Dijk[13], Marja C. Huizer[10,11,14], Christian V. Hulzebos[13], Ralph W.J. Leunissen[15], Beata Pazur[14], Ben J.P.W. Snoeren[16], Bente de Vries[17], Leo Wewerinke[18].

*Corresponding author: Jasper V. Been, Division of Neonatology, Department of Paediatrics, Erasmus MC – Sophia Children's Hospital, University Medical Centre, PO Box 2060, 3000 CB Rotterdam, Netherlands;

E: j.been@erasmusmc.nl; T: +31-10-7037048; F: +31-10-7036815.

[1]Department of Obstetrics and Gynaecology, Division of Obstetrics and Prenatal Medicine, Erasmus MC, University Medical Centre Rotterdam, the Netherlands.

[2]Department of Paediatrics, Division of Neonatology, Erasmus MC – Sophia Children's Hospital, University Medical Centre Rotterdam, the Netherlands.

[3]Institute for Medical Technology Assessment (iMTA), Erasmus University Rotterdam, Rotterdam, the Netherlands.

[4]Intensive Care and Department of Paediatric Surgery, Erasmus MC – Sophia Children's Hospital, University Medical Centre Rotterdam, The Netherlands.

For peer review only

For peer review only - http://bmjopen.bmj.com/site/about/guidelines.xhtml

[5]Department of Paediatrics, Intensive Care Unit, Erasmus MC – Sophia Children's Hospital, University Medical Centre Rotterdam, the Netherlands.

[6]Department of Internal Medicine, Nursing Science, Erasmus MC, University Medical Centre Rotterdam, the Netherlands

[7]Department of Public Health, Erasmus MC, University Medical Centre Rotterdam, the Netherlands.

[8]Department of Paediatrics, Maasstad Hospital, Rotterdam, the Netherlands.

[9]Department of Paediatrics, Isala – Amalia Children's Clinic, Zwolle, the Netherlands.

[10]Primary care birth centre Haga, The Hague, the Netherlands.

[11]Primary care birth centre Maasstad, Rotterdam, the Netherlands.

[12]Department of Paediatrics, Elisabeth-TweeSteden Hospital, Tilburg, the Netherlands.

[13]Department of Neonatology, University Medical Centre Groningen – Beatrix Children's Hospital, University of Groningen, Groningen, the Netherlands.

[14]Primary care birth centre Rotterdam Noord, Rotterdam, the Netherlands.

[15]Department of Paediatrics, Haaglanden Medical Centre Westeinde, The Hague, the Netherlands.

[16]Primary care birth centre Fam, Tilburg, the Netherlands.

[17]Primary care birth centre Westeinde, The Hague, the Netherlands.

[18]Department of Paediatrics, Haga Hospital – Juliana Children's Hospital, The Hague, the Netherlands.

Word count: 4248

For peer review only

ABSTRACT

Introduction:

Jaundice, caused by hyperbilirubinaemia, is a physiological phenomenon in the neonatal period. However, severe hyperbilirubinaemia – when left untreated – may cause kernicterus, a severe condition resulting in lifelong neurologic disabilities. Although commonly applied, visual inspection is ineffective in identifying severe hyperbilirubinaemia. We aim to investigate whether, among babies cared for in primary care: 1. transcutaneous bilirubinometry (TcB) screening can help reduce severe hyperbilirubinaemia, and 2. primary care-based (versus hospital-based) phototherapy can help reduce hospital admissions.

Methods and analysis:

A factorial stepped-wedge cluster randomised controlled trial will be conducted in seven Dutch primary care birth centres (PCBC). Neonates born after 35 weeks of gestation, cared for at a participating PCBC during at least two days within the first week of life are eligible, provided they have not received phototherapy before. According to the stepped-wedge design, following a phase of 'usual care' (visual assessment and selective total serum bilirubin (TSB) quantification), either daily TcB measurement or, if indicated, phototherapy in the PCBC will be implemented (phase 2). In phase 3, both interventions will be evaluated in each PCBC. We aim to include 5500 neonates over 3 years.

Primary outcomes are assessed at 14 days of life: 1. the proportion of neonates having experienced severe hyperbilirubinaemia (for the TcB screening intervention), defined as a TSB above the mean of the phototherapy and the exchange transfusion threshold, and 2. the proportion of neonates having required hospital admission for hyperbilirubinaemia treatment (for the phototherapy intervention in primary care).

Ethics and dissemination:

This study has been approved by the Medical Research Ethics Committee of the Erasmus MC Rotterdam, the Netherlands (MEC-2017-473). Written parental informed consent will be obtained.

For peer review only - http://bmjopen.bmj.com/site/about/guidelines.xhtml

Results from this study will be published in peer-reviewed journals and presented at (inter)national meetings.

Trial registration number: NTR7187 (Dutch Trial Registry)

Protocol date and version: 19 June 2018, version 3.1

Keywords: neonatal unconjugated hyperbilirubinemia, neonatal jaundice, kernicterus, transcutaneous bilirubin measurement, phototherapy, primary health care, birth center, randomized controlled trial, stepped wedge, cluster, factorial.

For peer review only

For peer review only - http://bmjopen.bmj.com/site/about/guidelines.xhtml

STRENGHTS AND LIMITATIONS OF THIS STUDY

- This study is the first randomised controlled trial to assess the (cost-)effectiveness of transcutaneous bilirubin measurement as a universal screening tool for potentially severe jaundice, addressing a knowledge gap highlighted in several national guidelines.
- This study is also the first randomised controlled trial to assess the (cost-)effectiveness of phototherapy outside the hospital setting.
- The stepped-wedge design of this study allows all participating primary care birth centres to have introduced both interventions as standard care towards the end of the study, which will aid implementation if (cost-)effectiveness of the interventions is demonstrated.
- A limitation is that parents and care providers cannot be blinded to allocation of the interventions.

For peer review only

For peer review only - http://bmjopen.bmj.com/site/about/guidelines.xhtml

INTRODUCTION

In newborns, severe hyperbilirubinaemia may cause brain damage when it is not recognised or is left untreated.[1,2] This spectrum of neurological sequelae is called kernicterus spectrum disorders (KSD) with acute bilirubin encephalopathy and its consequences at the extreme end and more subtle disorders at the other.[1,3] KSD also encompasses "classical kernicterus", a chronic, irreversible, neuropathological disorder involving neuromotor dysfunction, auditory disorders, and oculomotor impairments.[1-4]

In high income countries an estimated 1 in 67,000 neonates develops classical kernicterus.[5,6]

Timely recognition of potentially severe jaundice, caused by hyperbilirubinaemia, is essential to prevent KSD.[7,8] Traditionally, jaundice is identified via visual inspection by maternity care professionals, midwives or parents. If potentially severe jaundice is suspected, total serum bilirubin (TSB) is quantified in blood taken via a skin prick to assess the need for treatment.[9] However, visual inspection of neonatal jaundice is proven to be inaccurate in detecting hyperbilirubinaemia [10,11] and therefore ineffective in preventing kernicterus.[12]

Guidelines in several high-income countries advise universal screening for jaundice in newborns using TSB or transcutaneous bilirubin (TcB) measurement.[10,13] Several observational studies have shown a good correlation between TSB and TcB measurements in the neonatal period.[14,15] A programmatic evaluation of a TcB-based screening programme in Canada demonstrated a 55% reduction in severe hyperbilirubinaemia and related health care utilisation as compared to visual inspection with selective TSB quantification.[16] Similar findings were obtained in a retrospective multicentre study evaluating a screening program using standard versus selective TSB or TcB measurement in the United States.[17]

Universal screening for neonatal jaundice in the primary care setting has the potential to reduce the incidence of severe hyperbilirubinaemia.[2,18] This would also bring down the number of patients who require exchange transfusion, which is a highly invasive treatment that carries significant health risks and substantial costs.

For peer review only - http://bmjopen.bmj.com/site/about/guidelines.xhtml

Hyperbilirubinaemia is usually treated with phototherapy in a hospital setting, which may be applied via various modalities.[2] Fibreoptic phototherapy using a underneath device (i.e. mattress) is effective in reducing TSB levels, is safe,[19] and meets existing guidelines.[9] There is some evidence to suggest that fibreoptic phototherapy may safely and effectively be used in the primary care setting as well.[14,20,21] Institution of phototherapy in the primary care setting may reduce the number of neonates admitted to hospital for hyperbilirubinaemia treatment and reduce associated costs.[22]

Whereas universal TcB screening for neonatal hyperbilirubinaemia and phototherapy provided in primary care have the potential to be effective and cost-effective via reducing the incidence of severe hyperbilirubinaemia and the need for hospitalisation,[14,23] evidence to support these assertions is currently lacking, while in today's health care arena cost-effectiveness considerations are becoming increasingly important.

We hypothesise that non-invasive screening for neonatal hyperbilirubinaemia using TcB and application of phototherapy in primary care will (cost-)effectively reduce the incidence of severe hyperbilirubinaemia and the need for hospital admission for hyperbilirubinaemia treatment. Here, we present our protocol for a factorial stepped-wedge cluster randomised controlled trial (RCT) among newborns cared for in primary care to test these hypotheses.

METHODS AND ANALYSIS

Study design

We will conduct a factorial stepped-wedge cluster RCT in seven primary care birth centres (PCBCs) in the Netherlands to evaluate among newborns the effectiveness of: (1) screening for hyperbilirubinaemia using daily TcB quantification to reduce the incidence of severe hyperbilirubinaemia, and (2) treatment of hyperbilirubinaemia (if indicated) using phototherapy instituted within the PCBC to reduce the need for hospital admission. Accordingly, each PCBC will start

For peer review only - http://bmjopen.bmj.com/site/about/guidelines.xhtml

with (1) a control phase in which usual care is evaluated, followed by (2) a second phase in which one of both interventions is evaluated, followed by (3) a final phase in which both interventions are evaluated in parallel. More detail is provided below.

Dutch perinatal care system

In the Netherlands, a significant proportion of women (30% in 2016) give birth in a primary care setting (i.e. at home or in a PCBC) under supervision of a community midwife.[24] In addition, many who deliver in a hospital setting are discharged home or to a PCBC within 24 hours after delivery. Maternity care assistants provide maternity care when the neonate and the mother are at home or in a PCBC, under supervision of the community midwife (who will visit at least 3 times in the first week).[25] Only healthy neonates will be cared for at home or in a PCBC (i.e. neonates with congenital infection or other diseases will receive initial treatment in the hospital). The community midwife will consult the paediatrician of an affiliated nearby hospital if a potential clinical problem such as hyperbilirubinaemia presents in the neonate. Neonates with a gestational age above 35 weeks who have been discharged from the hospital are especially at risk of developing severe unconjugated hyperbilirubinaemia.[26]

Participants

Study sites

This study will be performed in seven PCBCs in the Netherlands. In these PCBCs altogether approximately 5,000 neonates and their mothers are admitted for at least 48 hours each year. Each of the five largest PCBCs will be considered a cluster in the context of our cluster RCT. The two smallest PCBCs will be paired and will form a cluster together. A PCBC was selected for the study if it facilitated provision of maternity care during the first days postpartum (that is, some PCBCs only facilitate deliveries and do not provide care beyond the first 24 hours after delivery).

Patient eligibility

For peer review only - http://bmjopen.bmj.com/site/about/guidelines.xhtml

For peer review only

Neonates are eligible for inclusion in the study if:

- The neonate was born after 35 completed weeks of gestation.

  Note: Only neonates with a gestational age of 37 weeks or higher are born in primary care; Nevertheless, a neonate may be eligible when born in the hospital between 35 and 37 weeks gestational age and then discharged to a PCBC within seven days after birth.
- The neonate is admitted to a participating PCBC within the first week of life.
- The neonate is expected to remain admitted for at least two days (to allow for serial TcB measurements to take place).
- Signed informed consent (IC) is provided by parent(s) or primary caregiver(s).

Neonates are not eligible if:

- The neonate received phototherapy previously or is currently receiving phototherapy (reliability of TcB measurement is reduced in neonates who are receiving or have received phototherapy).[14]
- Parents do not have sufficient understanding of the Dutch language to be able to comprehend the patient information sheet and questionnaire.

The enrolment of participants has started in July 2018 and is planned to be completed in July 2021.

Interventions and control

In this study, two interventions will be assessed in a 2x2 factorial design to determine the effectiveness of each intervention separately. Timing of implementation of each intervention will be allocated at the PCBC level.

Control group: usual care

During this initial study phase, usual care will be evaluated. Accordingly, visual inspection is used to identify jaundice, as advised in the Dutch national guideline for neonatal hyperbilirubinaemia.[9] When potentially severe jaundice is suspected, a skin prick will be performed to determine TSB. The TSB level

For peer review only - http://bmjopen.bmj.com/site/about/guidelines.xhtml

For peer review only

will be plotted on the Dutch nomogram [27] (which is similar to the nomogram of the American Academy of Pediatrics and explained in Text box 1) and be discussed with the affiliated paediatrician, according to local procedures. If treatment for hyperbilirubinaemia is indicated, as judged by the affiliated paediatrician, the neonate will be admitted to the hospital for this purpose.

Intervention 1: TcB measurement as a non-invasive screening tool

During the study phase in which this intervention is tested, TcB will be measured daily starting from 24 hours of age using the Dräger JM-105, a CE marked, and validated hand-held bilirubinometer, in all included neonates admitted to a PCBC allocated to this intervention.[28-31] If TcB exceeds the phototherapy threshold, as determined by the Dutch TSB nomogram for neonates born after 35 weeks of gestation [9], TSB will be quantified. Because the nomograms were originally designed to be applied to TSB measurements and because TcB measurements have an inaccuracy of maximally 50 µmol/L (i.e. 2.9 mg/dL),[14,32] TSB will also be quantified if TcB is less than 50 µmol/L below the phototherapy treatment threshold. The TSB level obtained will be discussed with an affiliated paediatrician (of the nearby hospital) in order to determine the need for phototherapy, again based on the Dutch nomogram.[9] A second TcB measurement on the same day may be performed at the discretion of the maternity care professional (e.g. if the TcB level is close to the threshold for measuring TSB).

Following institution of phototherapy, bilirubin levels will be monitored using TSB rather than TcB given the reduced reliability of TcB in neonates receiving or having received phototherapy.[14]

For peer review only

For peer review only - http://bmjopen.bmj.com/site/about/guidelines.xhtml

The Dutch TSB nomogram [27]

The Dutch TSB nomogram has the same values as the nomogram of the American Academy of Pediatrics. The nomogram consists of three risk categories: lower risk, medium risk and higher risk. The risk assessment is based on gestational age and several risk factors (blood group antagonism, other haemolytic disease, birth asphyxia, suspicion of infection/sepsis, the neonate being ill or drowsy, and serum albumin level below 30 g/L). The need for phototherapy and exchange transfusion depend on the risk assessment and the postnatal age of the neonate. A translated version of the Dutch TSB nomogram is depicted in Figure 1.

Figure 1: phototherapy and exchange transfusion thresholds for neonates with a gestational age of 35 weeks or more (TSB = total serum bilirubin; PT = phototherapy; ET = exchange transfusion; AS = Apgar score). Translated from Dutch. The Dutch nomogram can be downloaded from http://babyzietgeel.nl/kinderarts/hulpmiddelen/diagnostiek/bilicurve35wkn.php.

Text box 1: the Dutch TSB nomogram

review only

Intervention 2: phototherapy in the PCBC

During this study phase the default location for application of phototherapy – if clinically indicated – will be the PCBC, rather than the hospital. The indication for phototherapy is made by a paediatrician in the affiliated hospital based on the TSB level, according to the Dutch nomogram.[9] Phototherapy in the PCBC will be provided using the GE Healthcare BiliSoft Large, a commercially available, CE marked and validated phototherapy mattress using blue LED-light.[33] This phototherapy mattress is suitable for application in primary care as well as in the hospital setting.

The affiliated paediatrician will decide whether other blood tests have to be performed to determine the cause of hyperbilirubinaemia and at what time points follow-up TSB measurements need to be taken. Based on the results of these TSB measurements (dis)continuation of phototherapy and further follow-up is determined by the affiliated paediatrician. The affiliated paediatrician may decide to admit a neonate to the hospital for further hyperbilirubinaemia treatment at any time if there are strong

For peer review only - http://bmjopen.bmj.com/site/about/guidelines.xhtml

reasons to do so; these reasons will be recorded. Phototherapy will not be applied in the PCBC in neonates who have hyperbilirubinaemia above the phototherapy threshold within 24 hours after birth, since this is considered to represent a likely pathologic cause of hyperbilirubinaemia and bilirubin levels may rise swiftly in these neonates.[9]

Each PCBC will start with a time period without any intervention (control period), followed by a time period with only one of the interventions, and then a time period with both interventions. The inclusion of neonates will take 2.5 years per PCBC. The sequence and the timing at which the PCBC will switch over to the next intervention period will be determined by random allocation. The allocation scheme with the possible sequences of the interventions is depicted in Figure 2. The time blocks will take 4 months, except for the first and the last block. These two blocks will cover 5.5 months, because, to be able to make a comparison within a cluster, a sufficient number of participants in the control period (C) and in the period with both interventions (I1+I2) should be included, even when a smaller PCBC is randomised to time line 1 or 6.

Figure 2: allocation scheme (C = control; I1 = intervention 1: TcB measurement; I2 = intervention 2: phototherapy in PCBC)

A flowchart with an overview of the study procedures with the different intervention phases and possible orders of intervention phases from the perspective of a participant is displayed in Figure 3.

Figure 3: flowchart for study inclusion

Primary outcomes

The two interventions each have their own primary outcome. The primary outcome for TcB measurement as a screening tool for jaundice will be the proportion of neonates having severe

For peer review only - http://bmjopen.bmj.com/site/about/guidelines.xhtml

hyperbilirubinaemia at any time point within the first 14 days of life. Severe hyperbilirubinaemia is defined as a TSB level above the mean of the phototherapy and exchange transfusion threshold, according to the Dutch nomogram; [9] i.e.:

$$TSB > \frac{phototherapy\ threshold + exchange\ transfusion\ threshold}{2}$$

For the second intervention, phototherapy in the PCBC, the primary outcome will be the proportion of neonates requiring hospital admission for hyperbilirubinaemia treatment within the first 14 days of life. Hyperbilirubinaemia treatment will be considered the indication for hospital admission if the neonate received phototherapy or an exchange transfusion for hyperbilirubinaemia within 24 hours after hospital admission.

Secondary outcomes

The secondary outcomes will also be assessed within the first 14 days of life (except for kernicterus) and are listed in Table 1.

Table 1: secondary outcomes

Secondary outcomes		
**Assessed in**	**Outcome**	**Recorded by**
**All neonates**	Number of times TcB quantified	Maternity care professional
	Individual TcB readings	Maternity care professional
	Highest TSB level	Maternity care professional and/or EMR in hospital
	Number of neonates having TSB quantified	Maternity care professional
	Number of times blood taken for TSB quantification	Maternity care professional and/or EMR in hospital
	Individual TSB levels	Maternity care professional and/or EMR in hospital

For peer review only - http://bmjopen.bmj.com/site/about/guidelines.xhtml

	Number of times blood taken for TSB quantification before start of phototherapy	Maternity care professional and/or EMR in hospital
	Number of neonates receiving phototherapy	Maternity care professional and/or EMR in hospital
	Duration (hours) of phototherapy	Maternity care professional and/or EMR in hospital
	Number of neonates having a TSB level above exchange transfusion threshold [9]	Maternity care professional and/or EMR in hospital
	Number of neonates who actually received an exchange transfusion	EMR in hospital
	Number of neonates having kernicterus*	EMR in hospital and/or EMR at general practitioner
	Duration of stay in the PCBC	Maternity care professional
	Duration of hospital stay following initial PCBC admission (if relevant)	EMR in hospital
	Number of transfers between PCBCs/hospitals	Maternity care professional and parental questionnaire
	Number of times affiliated paediatrician consulted	Maternity care professional
	Experience of parents regarding hyperbilirubinaemia assessment and treatment	Parental questionnaire
	Experience of attending maternity care personnel regarding the daily practice of hyperbilirubinaemia assessment and treatment during control and intervention periods, including facilitators and barriers for implementation	Maternity care assistant questionnaire
**Within the group included in the intervention period with phototherapy in the PCBC**	The number of neonates in whom phototherapy was initiated in the hospital, including reasons for this hospital admission	EMR in hospital
	The number of neonates requiring subsequent hospital admission for hyperbilirubinaemia treatment (i.e. after initiation of phototherapy in the PCBC), including reasons for this 'treatment failure'	EMR in hospital

EMR = electronic medical record

For peer review only - http://bmjopen.bmj.com/site/about/guidelines.xhtml

For peer review only

*The diagnosis of kernicterus will be made by combining clinical signs and symptoms and additional investigations (e.g. cerebral ultrasound, MRI) up to one year after birth in every neonate who had a TSB level above the exchange transfusion threshold level.

Cost effectiveness analysis

A cost effectiveness analysis (CEA) will be performed from the health care perspective. In the CEA the costs and outcomes of the interventions, compared to 'usual care', will be identified, measured, and valued.

For all of the following items, health care use will be measured: TSB and TcB tests, phototherapy in the hospital, phototherapy in the PCBC, exchange transfusions, PCBC and/or hospital admission days, and ambulance transportation to a hospital. These data will be recorded during the study and/or retrieved from the electronic information systems of the participating PCBCs and affiliated hospitals.

Then, integral unit prices will be calculated using real economic cost prices or using standard cost-prices for health economic evaluations.[34] Unit prices will be multiplied by the quantities for each resource used and summed over the separate types of resource to give a total cost per patient.

Regarding the outcomes, the CEA will look at the number of neonates with severe hyperbilirubinaemia (as defined above) and the number of hospital admissions. Data on costs and outcomes will be measured throughout the 14-day observation period. As the final outcome measure of the CEA, incremental cost effectiveness ratios (ICERs) will calculated, expressed as incremental costs per case of severe hyperbilirubinaemia or hospitalisation avoided. Uncertainty in the estimation of the ICERs will be illustrated through cost-effectiveness planes. Where relevant, sensitivity analyses will be performed to assess the robustness of the analyses to certain assumptions.

Sample size

Two sample size calculations are required since two interventions, each with their own outcome, are evaluated. The interventions are considered to be independent of each other and therefore the largest sample size among the two sample size calculations is considered appropriate for the entire study. Sample size calculations are based on detecting superiority rather than non-inferiority.

For peer review only - http://bmjopen.bmj.com/site/about/guidelines.xhtml

For peer review only

The estimated incidence of severe hyperbilirubinaemia in term neonates in the Netherlands is 1.2% per year.[9] A previous study in Canada regarding screening for unconjugated hyperbilirubinaemia using transcutaneous bilirubinometry among healthy neonates ≥35 weeks of gestation showed a relative reduction in severe hyperbilirubinaemia of 55% following implementation.[16] We therefore hypothesise that the TcB screening intervention will decrease the incidence of severe hyperbilirubinaemia from 1.2% to 0.5% (i.e. by 0.7% points; a relative reduction of 58%). At a power of 80% and a two-sided alpha of 0.05, 2691 neonates per arm are needed to identify this effect.

The estimated proportion of neonates needing phototherapy is 4%.[9] We anticipate that approximately 50% of the neonates with hyperbilirubinaemia will have an indication to be admitted to the hospital (e.g. feeding difficulties in addition to hyperbilirubinaemia or need for intensive phototherapy). Therefore, we hypothesise that the planned application of phototherapy in the PCBC will decrease the need for hospital admission for hyperbilirubinaemia by 2% points (i.e. 50% relative reduction). At a power of 80% and a two-sided alpha of 0.05, 1136 neonates per arm are needed to identify this effect. While our study is a cluster RCT, given the cross-sectional nature of a stepped-wedge design the effect of intracluster correlation on power is minimal, therefore we did not adjust for intracluster correlation.[35]

Based on the largest sample size of 2691 neonates per arm, 5382 neonates are needed in total. We aim to include 5500 neonates to account for a degree of missing data.

Randomisation and blinding

Each of the participating PCBCs will be randomised to one of the predefined timelines (as depicted in Figure 2 above) by an online randomisation module (www.randomization.com). The two smallest PCBCs will be paired and randomised to the same timeline (see Figure 2).

For peer review only - http://bmjopen.bmj.com/site/about/guidelines.xhtml

Given the nature of the treatment in the intervention groups and the control group, blinding of (parents of) participants and health care personnel is not possible. Analyses will be performed by a researcher who is blinded to the PCBC allocation scheme.

Study procedures

All PCBCs in the Netherlands that provide maternity care to mothers and neonates in the first days after delivery were invited to participate. Eligibility of neonates admitted to the PCBC will be assessed by the maternity care or study personnel based on the inclusion and exclusion criteria. Informed consent of parents will be obtained by the maternity care professional. Research nurses and study personnel will support the maternity care professionals in obtaining informed consent and collecting data.

Depending on the allocation and the moment of admission in the PCBC during the study period, the neonate is included in the control period, in a time period where one of the interventions is implemented, or in a time period where both interventions are implemented. Standard operating procedures (SOPs) for each study phase have been developed.

Maternity care personnel of all PCBCs will be trained in the SOPs and in using the transcutaneous bilirubinometer and the phototherapy mattress to ensure the interventions are applied in a professional standardised manner. For this purpose, an e-learning module has been developed (available from: https://xoteur.12change.eu/play.php?template_id=798); in addition, during the study period three training sessions for maternity care professionals are held at each PCBC prior to the start of each subsequent study phase (i.e. intervention implementation).

Data collection

For peer review only - http://bmjopen.bmj.com/site/about/guidelines.xhtml

Data collection at the PCBC will be performed by maternity care personnel. Together with the training for using the transcutaneous bilirubinometer and the phototherapy mattress, maternity care personnel will be trained in adequate data collection. Data collection is described in the SOPs as well.

After inclusion, a maternity care professional of the PCBC will record baseline characteristics of the mother and the neonate (see Table 2) using a standardised registration form at a secured internet page. All participants will receive an anonymised study number.

The maternity care professionals will record several measurements during PCBC stay. These measurements are noted in Table 3. All data will be recorded in standardised case report forms. The study team, the study monitor, and (inter)national supervisory authorities will have access to the data and the final dataset.

Table 2: baseline characteristics

**Baseline characteristics**	
**Parental characteristics**	Maternal age
	Maternal ethnicity
	Maternal blood group
	Paternal ethnicity
	Presence of haemolytic disease (other than blood group antagonism) in mother or father
**Characteristics of pregnancy and birth**	Parity
	Maternal atypical red-cell alloantibodies during pregnancy
	Gestational age at birth
	Type of delivery: vaginal delivery, vacuum-assisted vaginal delivery, forceps-assisted vaginal delivery, caesarean delivery without vacuum or forceps extraction (before or during delivery), caesarean delivery with vacuum or forceps extraction (before or during delivery).
**Neonatal characteristics**	Date of birth
	Time of birth
	Sex
	Birth weight (in grams)
	Presence of birth trauma

For peer review only - http://bmjopen.bmj.com/site/about/guidelines.xhtml

	Type of feeding
	Neonatal blood group (if known)
	Foetal or neonatal Rhesus factor (if known)
	Direct antiglobulin (Coombs) test (if known)
	Presence of haemolytic disease (other than blood group antagonism)
	Presence of birth asphyxia: Apgar score <5 at 5 minutes or pH <7.0 in umbilical cord blood
	Suspicion of sepsis
	Neonate being ill or drowsy
**Other characteristics**	Siblings who experienced neonatal hyperbilirubinaemia (and cause of hyperbilirubinaemia, if known)

Table 3: daily measurements in PCBC

**Daily measurements**	
Control period	Skin colour: pink, slightly yellow, moderately yellow, quite yellow, very yellow
	Weight (in grams)
	Risk factors for hyperbilirubinaemia (if present)
	TSB values with date and time of measurement (if relevant)
	Decisions made based on TSB (if relevant)
Period with TcB screening (extra measurements in addition to control period)	TcB values measured at forehead and sternum together with date and time of measurement (once a day or two times if indicated)
Phototherapy in PCBC (extra measurements in addition to control period)	Start and end date and time of phototherapy
	Decisions made by the affiliated paediatrician regarding phototherapy

A questionnaire for the parents/caregivers to evaluate their experience will be sent by e-mail 14 days after birth of the neonate. This questionnaire will assess parental satisfaction, burdensomeness of interventions for the neonate, and experienced competence of maternity care professionals. If parents do not have access to the internet or do not respond to the questionnaire by e-mail, they will be approached by regular mail or by phone. The community midwife and/or the general practitioner will be approached if parents do not respond at all. Both after the control and after each intervention period, maternity care personnel will be invited to complete a questionnaire about their experiences regarding the daily practice of hyperbilirubinaemia assessment and treatment during these periods

For peer review only - http://bmjopen.bmj.com/site/about/guidelines.xhtml

(e.g. facilitators and barriers for implementation, sense of competence regarding applying the new interventions, integrating the new interventions in routine care, and the content of the training regarding the new interventions).

Statistical analysis

We will use cluster specific methods because randomisation will be performed at the PCBC level rather than at the individual level. For both primary objectives an intention-to-treat analysis will be performed.

To evaluate whether the occurrence of the primary outcomes of both interventions are different between the study periods with and without the intervention(s), generalised linear mixed models (GLMMs) will be used. GLMMs account for the systematically different observation periods and for clustering in the data.[36] In case of missing data, multiple imputation using chained equations will be used. Relative risks, risk differences, and relative risk reduction with corresponding 95% confidence intervals will be calculated from the GLMMs.

Subgroup analyses to evaluate the effect of the interventions in neonates of different ethnicities (Caucasian and non-Caucasian) and different gestational age groups (<38 weeks of gestation and ≥38 weeks of gestation) will be performed.

We will perform a per-protocol analysis to assess the impact of potential contamination during the first days of a new intervention period as a sensitivity analysis in addition to the intention-to-treat analysis.

Safety

Adverse events noticed by parents or maternity care professionals will be recorded in the case report forms by the maternity care assistant or study personnel. Serious adverse events will be communicated to the study team within four days. The study team will investigate this event and report it to the

For peer review only - http://bmjopen.bmj.com/site/about/guidelines.xhtml

sponsor and the Medical Research Ethics Committee of the Erasmus MC Rotterdam. Insurance has been taken out for every participant in this study. The insurance covers any damage caused by the study.

The medical technical service department of the Erasmus MC-Sophia will provide technical maintenance of the phototherapy mattresses (at least two times per year). Once a year, calibration of the TcB devices will be carried out by the manufacturer.

A Data Safety Monitoring Board will not be established, given that the risks of this study are considered negligible. Monitoring of the study will be carried out according to the Guideline for Good Clinical Practice by an independent, qualified monitor at least once a year per PCBC.[37]

Patient and public involvement

The study protocol, study procedures and patient information form were discussed with the regional patient involvement board of the Rijnmond Obstetric District Platform ('District Verloskundig Platform'; DVP) and patient representatives of the Child & Hospital foundation ('Stichting Kind & Ziekenhuis': https://www.kindenziekenhuis.nl/). They consider the project highly relevant from a patient perspective and have agreed to be involved throughout execution, analysis and interpretation, dissemination to patients, and implementation. The involved patient panels have considered the burden of the interventions negligible and underline the potential benefits of the study.

ETHICS AND DISSEMINATION

This study has been approved by the Medical Research Ethics Committee of the Erasmus MC Rotterdam (MEC-2017-473). Collected data will be coded. Only investigators will have access to identification list. Study documents and data will be stored for 15 years after completion of the study. Written informed consent is provided by parents of neonates included in the study. The study is registered in the Dutch Trial Registry (NTR7187).

For peer review only - http://bmjopen.bmj.com/site/about/guidelines.xhtml

The full protocol will be shared at request. Results of this study including the statistical code will be published in peer-reviewed scientific journals and be presented at (inter)national meetings. The results of the study will be reported according to the Consolidated Standards of Reporting Trials (CONSORT) Statement guidelines.[38] We intend to provide an anonymised version of the dataset at request. Authorship will be determined according to the guidelines of the International Committee of Medical Journal Editors (ICMJE).[39] No professional writers will be used.

DISCUSSION

Identification and (referral for) treatment of neonatal jaundice is daily practice for maternity care professionals. The continuing occurrence and devastating consequences of KSD underline the urgent need for more effective identification and treatment of at-risk neonates.[1,7] This pragmatic study aims to evaluate the (cost-)effectiveness of (1) TcB measurement as a screening tool for jaundice in a primary care setting and (2) application of phototherapy in the PCBCs in the Netherlands. To the best of our knowledge, this RCT will be the first to assess the (cost-)effectiveness of these diagnostic and treatment modalities in a primary care setting.

The study design allows all participating PCBCs to implement both interventions during the study and as such all PCBCs will have implemented the interventions as standard care toward the end of the study. This process will provide valuable information about the facilitators and barriers for implementation that will help upscaling of the interventions if (cost-)effectiveness is confirmed.

A limitation of this study is that parents and care providers cannot practically be blinded to allocation of the interventions. As a result, maternity care personnel or paediatricians may act differently in control and intervention periods. We will try to identify this via the questionnaires addressed at the maternity care assistants and the recording of the underlying reasons for paediatricians to divert from the new practice of applying phototherapy in the PCBC, if relevant. We will furthermore record the

For peer review only - http://bmjopen.bmj.com/site/about/guidelines.xhtml

number of times TSB is quantified to evaluate whether this may change following implementation of the screening programme.

For this study we take advantage of the fact that neonates receive primary care within PCBCs rather than at home. This centralised primary care provision is efficient in terms of feasibility of training the maternity care professionals and of minimising costs of transcutaneous bilirubinometers and phototherapy mattresses (i.e. groups of neonates can be covered with individual devices). Moreover, although the Dutch perinatal health care system is quite unique, care provision in PCBCs in the Netherlands is comparable to regular maternity care in many other high-income countries, which promotes generalisability.

We expect the interventions to be similarly effective in the home setting as in the PCBC setting, since the population in the PCBCs in the Netherlands does not differ from that the home setting. The costs in the PCBC setting may however differ from the home setting. Generalisability of the results to other countries and the home setting depends on several aspects, such as characteristics of the population, organisation of the maternity care system, presence of an effective screening program for neonatal hyperbilirubinaemia, and proximity and availability of sufficient health care. A future study to investigate the feasibility and effectiveness of the interventions in the home setting is needed if the current study is successful.

In conclusion, our study will generate useful data about the (cost-)effectiveness of TcB measurement as a universal screening tool for jaundice, potentially reducing the incidence of severe neonatal hyperbilirubinaemia. Several guidelines have highlighted the lack of research into the prevention of severe hyperbilirubinaemia.[9,10,13,40] Although most of these guidelines do not specify where phototherapy should be instituted, it is standard practice in most high-income countries – including the Netherlands – to do so in the hospital setting.[9,10,40] As such, institution of phototherapy in the primary care setting has significant potential to reduce health care costs associated with hospital

For peer review only - http://bmjopen.bmj.com/site/about/guidelines.xhtml

admission. Furthermore, it promotes mother-child bonding by allowing mother and child to remain together in the same location.

ACKNOWLEDGEMENTS

We acknowledge The Netherlands Organisation for Health Research and Development and Erasmus MC for their financial support to this research. We thank Rogier de Jonge for his support concerning the design and funding of this study, Verena Werdmüller von Elgg for her support regarding Medical Research Ethics Committee approval, Liesbeth Dusink and Roel Faber for their support regarding preparation for data collection and composing case report forms, Marc Sylva for help with literature review, Nina Witt, Rik Bouman and Jetty van Ginkel for their support regarding legal issues and contact with manufacturers, Imke Theeuwen for support during the training sessions, and other practical issues in the phase before the start of the study. We thank the patient involvement board of the Rijnmond Obstetric District Platform and the patient representatives of the Child & Hospital Foundation for their feedback regarding the patient perspective. We are grateful to the participating PCBCs, maternity care professionals, midwives, and affiliated paediatricians for taking part in this study.

AUTHORS' CONTRIBUTIONS

BAMG and JVB wrote the first version of the manuscript. JVB, JPG, RFK, IKMR and EAPS designed the study and secured funding. JVB and LCMB developed the methodological part of this study. MJP designed the cost-effectiveness analysis. EI designed evaluation of implementation of both interventions. All authors critically revised the manuscript and gave final approval of the version to be published.

FUNDING

For peer review only - http://bmjopen.bmj.com/site/about/guidelines.xhtml

This work is supported by a Health Care Efficiency Research grant of The Netherlands Organisation for Health Research and Development (ZonMw), grant number 843002805, and an Erasmus MC Efficiency Research grant, grant number 2016-16107. The study funders were not involved in the collection, management, analysis and interpretation of data; writing of the report; or the decision to submit the report for publication.

COMPETING INTERESTS

All authors declare that they do not have any competing interests.

For peer review only

REFERENCES

1. Shapiro SM. Definition of the clinical spectrum of kernicterus and bilirubin-induced neurologic dysfunction (BIND). *J Perinatol*. 2005;25(1):54-9.
2. Olusanya BO, Kaplan M, Hansen TWR. Neonatal hyperbilirubinaemia: a global perspective. *The Lancet Child & Adolesc Health*. 2018;2(8):610-20.
3. Bhutani VK, Johnson-Hamerman L. The clinical syndrome of bilirubin-induced neurologic dysfunction. *Semin Fetal Neonatal Med*. 2015;20(1):6-13.
4. Le Pichon JB, Riordan SM, Watchko J, et al. The Neurological Sequelae of Neonatal Hyperbilirubinemia: Definitions, Diagnosis and Treatment of the Kernicterus Spectrum Disorders (KSDs). *Curr Pediatr Rev*. 2017;13(3):199-209.
5. Slusher TM, Zamora TG, Appiah D, et al. Burden of severe neonatal jaundice: a systematic review and meta-analysis. *BMJ Paediatr Open*. 2017;1(1):e000105.
6. Sgro M, Campbell DM, Kandasamy S, et al. Incidence of chronic bilirubin encephalopathy in Canada, 2007-2008. *Pediatrics*. 2012;130(4):e886-90.
7. Bousema S, Govaert P, Dudink J, et al. [Kernicterus is preventable but still occurs] Kernicterus is vermijdbaar, maar komt nog steeds voor. *Ned Tijdschr Geneeskd*. 2015;159:A8518.
8. Sgro M, Campbell D, Shah V. Incidence and causes of severe neonatal hyperbilirubinemia in Canada. *CMAJ*. 2006;175(6):587-90.
9. Nederlandse Vereniging voor Kindergeneeskunde, Kwaliteitsinstituut voor de Gezondheidszorg CBO. [Guideline prevention, diagnosis and treatment of hyperbilirubinaemia among newborns born at a gestational age of more than 35 weeks] Richtlijn preventie, diagnostiek en behandeling van hyperbilirubinemie bij de pasgeborene, geboren na een zwangerschapsduur van meer dan 35 weken. 2008; Available from: https://www.nvk.nl/Portals/0/richtlijnen/hyperbili/richtlijnhyperbili.pdf.
10. Barrington KJ, Sankaran K, Canadian Paediatric Society, Fetus and Newborn Committee. Guidelines for detection, management and prevention of hyperbilirubinemia in term and late preterm newborn infants. *Paediatr Child Health*. 2007;12(Suppl B):1B-12B.
11. Maisels MJ, Clune S, Coleman K, et al. The natural history of jaundice in predominantly breastfed infants. *Pediatrics*. 2014;134(2):e340-5.
12. Lease M, Whalen B. Assessing jaundice in infants of 35-week gestation and greater. *Curr Opin Pediatr*. 2010;22(3):352-65.
13. American Academy of Pediatrics, Subcommittee on Hyperbilirubinemia. Management of Hyperbilirubinemia in the Newborn Infant 35 or More Weeks of Gestation. *Pediatrics*. 2004;114:297-316.
14. Bhardwaj K, Locke T, Biringer A, et al. Newborn bilirubin screening for preventing severe hyperbilirubinemia and bilirubin encephalopathy: a rapid review. *Curr Pediatr Rev*. 2017 Jan 10;13(1):67-90.
15. Engle WD, Jackson GL, Engle NG. Transcutaneous bilirubinometry. *Semin Perinatol*. 2014;38(7):438-51.
16. Wainer S, Parmar SM, Allegro D, et al. Impact of a transcutaneous bilirubinometry program on resource utilization and severe hyperbilirubinemia. *Pediatrics*. 2012;129(1):77-86.
17. Eggert LD, Wiedmeier SE, Wilson J, et al. The effect of instituting a prehospital-discharge newborn bilirubin screening program in an 18-hospital health system. *Pediatrics*. 2006;117(5):e855-62.
18. Bhutani VK, Johnson LH, Jeffrey Maisels M, et al. Kernicterus: epidemiological strategies for its prevention through systems-based approaches. *J Perinatol*. 2004;24(10):650-62.
19. Kumar P, Chawla D, Deorari A. Light-emitting diode phototherapy for unconjugated hyperbilirubinaemia in neonates. *Cochrane Database Syst Rev*. 2011 Dec;(12):CD007969.
20. Yilmaz A, Ozkiraz S, Akcan AB, et al. Low-cost home-use light-emitting-diode phototherapy as an alternative to conventional methods. *J Trop Pediatr*. 2015;61(2):113-8.
21. Walls M, Wright A, Fowlie P, et al. Home phototherapy in the United Kingdom. *Arch Dis Child Fetal Neonatal Ed*. 2004;89(3):F282.

For peer review only - http://bmjopen.bmj.com/site/about/guidelines.xhtml

22. Battersby C, Michaelides S, Upton M, et al. Term admissions to neonatal units in England: a role for transitional care? A retrospective cohort study. *BMJ Open*. 2017;7(5):e016050.
23. McClean S, Baerg K, Smith-Fehr J, et al. Cost savings with transcutaneous screening versus total serum bilirubin measurement for newborn jaundice in hospital and community settings: a cost-minimization analysis. *CMAJ Open*. 2018;6(3):E285-91.
24. Perined. [Perinatal Care in the Netherlands 2016] Perinatale Zorg in Nederland 2016. Utrecht: Perined, 2018.
25. de Boer J, Zondag L. [Multidisciplinary guideline postnatal care] Multidisciplinaire richtlijn Postnatale Zorg - Verloskundige basiszorg voor moeder en kind. Koninklijke Nederlandse Organisatie van Verloskundigen; 2018; Available from: https://www.knov.nl/serve/file/knov.nl/knov_downloads/2882/file/Postnatale_zorg_opgemaakte_versie_door_IB_md_10_aug_2018.pdf.
26. Gotink MJ, Benders MJ, Lavrijsen SW, et al. Severe neonatal hyperbilirubinemia in the Netherlands. *Neonatology*. 2013;104(2):137-42.
27. Dijk PH, de Vries TW, de Beer JJ. [Guideline 'Prevention, diagnosis and treatment of hyperbilirubinemia in the neonate with a gestational age of 35 or more weeks'] Richtlijn 'Preventie, diagnostiek en behandeling bij de pasgeborene, geboren na een zwangerschapsduur van meer dan 35 weken'. *Ned Tijdschr Geneeskd*. 2009;153:A93.
28. Raimondi F, Lama S, Landolfo F, et al. Measuring transcutaneous bilirubin: a comparative analysis of three devices on a multiracial population. *BMC Pediatr*. 2012;12:70.
29. Nagar G, Vandermeer B, Campbell S, et al. Reliability of transcutaneous bilirubin devices in preterm infants: a systematic review. *Pediatrics*. 2013;132(5):871-81.
30. Jones DF, McRea AR, Knowles JD, et al. A Prospective Comparison of Transcutaneous and Serum Bilirubin Within Brief Time Intervals. *Clin Pediatr (Phila)*. 2017;56(11):1013-7.
31. Romagnoli C, Zecca E, Catenazzi P, et al. Transcutaneous bilirubin measurement: comparison of Respironics BiliCheck and JM-103 in a normal newborn population. *Clin Biochem*. 2012;45(9):659-62.
32. Maisels MJ. Transcutaneous bilirubinometry. *Neoreviews*. 2006;7(5):e217-e25.
33. Bhutani VK. Phototherapy to prevent severe neonatal hyperbilirubinemia in the newborn infant 35 or more weeks of gestation. *Pediatrics*. 2011:e1046-52.
34. Hakkaart-van Roijen L, van der Linden N, Bouwmans C, et al. [Costing manual: Methodology of costing research and reference prices for economic evaluation in health care] Kostenhandleiding: Methodologie van kostenonderzoek en referentieprijzen voor economische evaluaties in de gezondheidszorg2015.
35. de Hoop E, van der Tweel I, van der Graaf R, et al. The need to balance merits and limitations from different disciplines when considering the stepped wedge cluster randomized trial design. *BMC Med Res Methodol*. 2015;15:93.
36. Hemming K, Haines TP, Chilton PJ, et al. The stepped wedge cluster randomised trial: rationale, design, analysis, and reporting. *BMJ*. 2015;350.
37. International Council for Harmonisation of Technical Requirements for Pharmaceuticals for Human Use (ICH). Guideline for Good Clinical Practice ICH. 2016.
38. Hemming K, Taljaard M, McKenzie JE, et al. Reporting of stepped wedge cluster randomised trials: extension of the CONSORT 2010 statement with explanation and elaboration. *BMJ*. 2018;363:k1614.
39. International Committee of Medical Journal Editors. Defining the Role of Authors and Contributors. 2017 [cited 2018 November 22]; Available from: http://www.icmje.org/recommendations/browse/roles-and-responsibilities/defining-the-role-of-authors-and-contributors.html.
40. National Collaborating Centre for Women's and Children's Health. Neonatal jaundice. National Institute for Health and Clinical Excellence; 2010; Available from: https://www.ncbi.nlm.nih.gov/pubmedhealth/PMH0016565/pdf/PubMedHealth_PMH0016565.pdf.

For peer review only - http://bmjopen.bmj.com/site/about/guidelines.xhtml

For peer review only

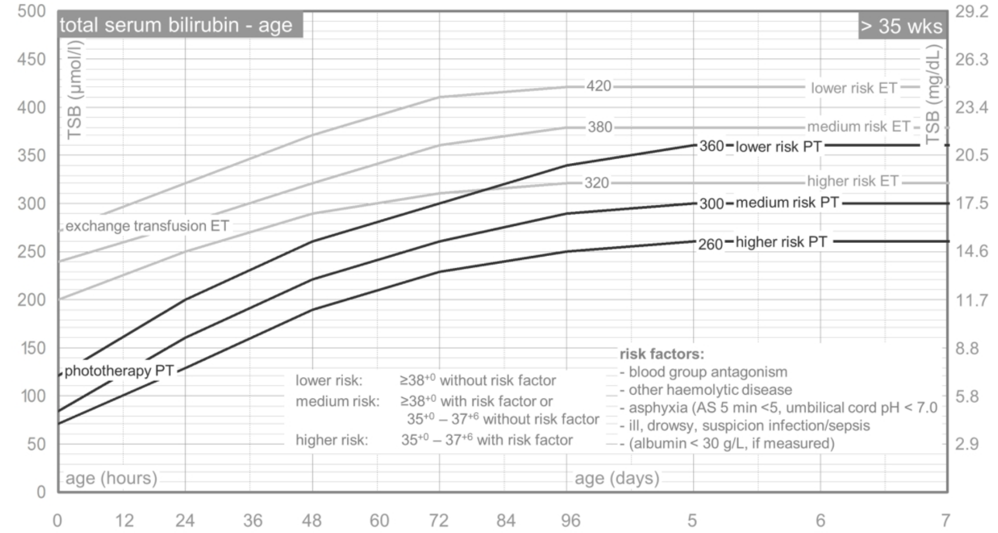

Figure 1: phototherapy and exchange transfusion thresholds for neonates with a gestational age of 35 weeks or more (TSB = total serum bilirubin; PT = phototherapy; ET = exchange transfusion; AS = Apgar score). Translated from Dutch.
The Dutch nomogram can be downloaded from http://babyzietgeel.nl/kinderarts/hulpmiddelen/diagnostiek/bilicurve35wkn.php.

173x99mm (300 x 300 DPI)

Cluster	Time →						
1	C	I1	I1	I1	I1	I1	I1 + I2
2	C	C	I1	I1	I1	I1 + I2	I1 + I2
3	C	C	C	I1	I1 + I2	I1 + I2	I1 + I2
4	C	C	C	I2	I1 + I2	I1 + I2	I1 + I2
5	C	C	I2	I2	I2	I1 + I2	I1 + I2
6	C	I2	I2	I2	I2	I2	I1 + I2

Figure 2: allocation scheme (C = control; I1 = intervention 1: TcB measurement; I2 = intervention 2: phototherapy in PCBC)

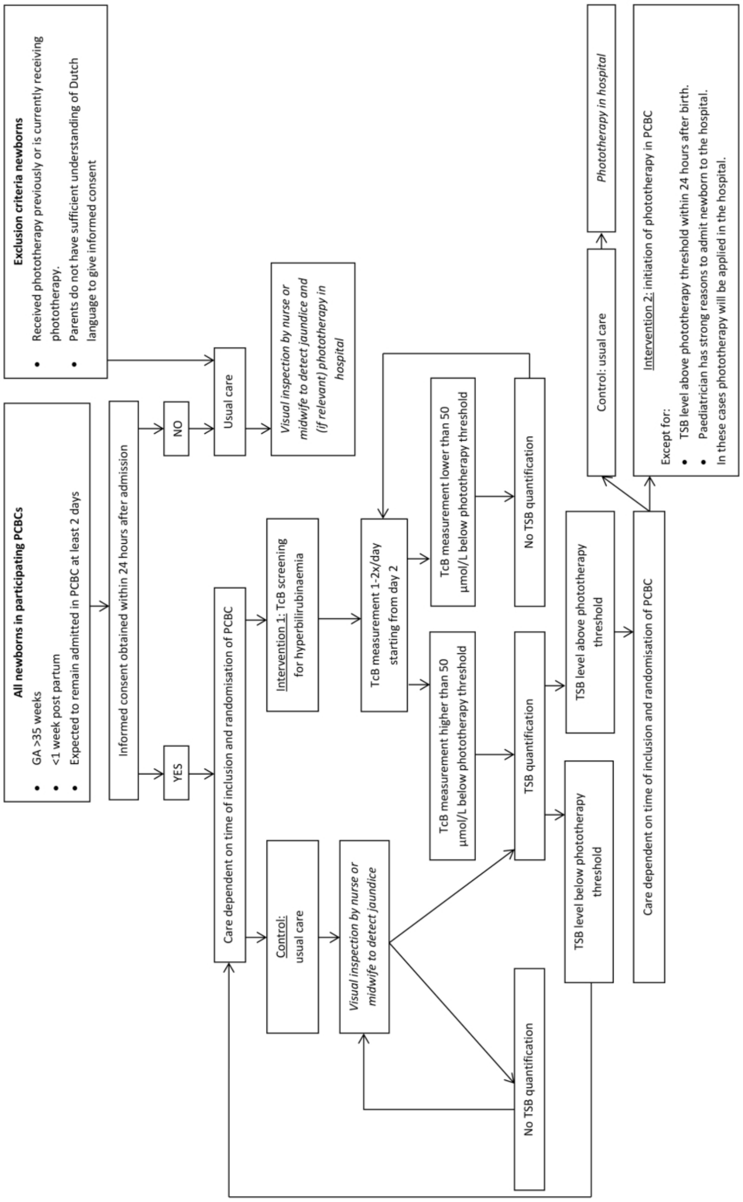

Figure 3: flowchart for study inclusion

146x233mm (300 x 300 DPI)

SPIRIT — Standard Protocol Items: Recommendations for Interventional Trials

SPIRIT 2013 Checklist: Recommended items to address in a clinical trial protocol and related documents*

Section/item	Item No	Description
**Administrative information**		
Title	1	Descriptive title identifying the study design, population, interventions, and, if applicable, trial acronym **see page 1**
Trial registration	2a	Trial identifier and registry name. If not yet registered, name of intended registry **see page 4 'Trial registration number' and page 23 'Ethics and dissemination'**
	2b	All items from the World Health Organization Trial Registration Data Set **see http://apps.who.int/trialsearch/Trial2.aspx?TrialID=NTR7187**
Protocol version	3	Date and version identifier **see page 4 'Protocol version and date'**
Funding	4	Sources and types of financial, material, and other support **see page 25 'Funding'**
Roles and responsibilities	5a	Names, affiliations, and roles of protocol contributors **see page 1 and page 25 'Authors' contributions'**
	5b	Name and contact information for the trial sponsor **see page 1 'Corresponding author'**
	5c	Role of study sponsor and funders, if any, in study design; collection, management, analysis, and interpretation of data; writing of the report; and the decision to submit the report for publication, including whether they will have ultimate authority over any of these activities **see page 25 'Funding'**
	5d	Composition, roles, and responsibilities of the coordinating centre, steering committee, endpoint adjudication committee, data management team, and other individuals or groups overseeing the trial, if applicable (see Item 21a for data monitoring committee) **n/a**
**Introduction**		
Background and rationale	6a	Description of research question and justification for undertaking the trial, including summary of relevant studies (published and unpublished) examining benefits and harms for each intervention **see page 6-7 'Introduction'**

For peer review only

	6b	Explanation for choice of comparators **see page 6-7 'Introduction'**
Objectives	7	Specific objectives or hypotheses **see page 7 'Introduction'**
Trial design	8	Description of trial design including type of trial (eg, parallel group, crossover, factorial, single group), allocation ratio, and framework (eg, superiority, equivalence, noninferiority, exploratory) **see page 7-8 'Study design'**

Methods: Participants, interventions, and outcomes

Study setting	9	Description of study settings (eg, community clinic, academic hospital) and list of countries where data will be collected. Reference to where list of study sites can be obtained **see page 8 'Dutch perinatal care system' and 'participants'**
Eligibility criteria	10	Inclusion and exclusion criteria for participants. If applicable, eligibility criteria for study centres and individuals who will perform the interventions (eg, surgeons, psychotherapists) **see page 9 'Patient eligibility'**
Interventions	11a	Interventions for each group with sufficient detail to allow replication, including how and when they will be administered **see page 9-14 'Methods'**
	11b	Criteria for discontinuing or modifying allocated interventions for a given trial participant (eg, drug dose change in response to harms, participant request, or improving/worsening disease) **see page 12 'Intervention 2: phototherapy in the PCBC'**
	11c	Strategies to improve adherence to intervention protocols, and any procedures for monitoring adherence (eg, drug tablet return, laboratory tests) **see page 19 'Study procedures'**
	11d	Relevant concomitant care and interventions that are permitted or prohibited during the trial **n/a**
Outcomes	12	Primary, secondary, and other outcomes, including the specific measurement variable (eg, systolic blood pressure), analysis metric (eg, change from baseline, final value, time to event), method of aggregation (eg, median, proportion), and time point for each outcome. Explanation of the clinical relevance of chosen efficacy and harm outcomes is strongly recommended **see page 15-17 and page 22 'Methods'**
Participant timeline	13	Time schedule of enrolment, interventions (including any run-ins and washouts), assessments, and visits for participants. A schematic diagram is highly recommended (see Figure) **see page 12-14 'Methods'**

For peer review only - http://bmjopen.bmj.com/site/about/guidelines.xhtml

Sample size	14	Estimated number of participants needed to achieve study objectives and how it was determined, including clinical and statistical assumptions supporting any sample size calculations **see page 17-18 'Sample size'**
Recruitment	15	Strategies for achieving adequate participant enrolment to reach target sample size **see page 19 'Study procedures'**

Methods: Assignment of interventions (for controlled trials)

Allocation:

Sequence generation	16a	Method of generating the allocation sequence (eg, computer-generated random numbers), and list of any factors for stratification. To reduce predictability of a random sequence, details of any planned restriction (eg, blocking) should be provided in a separate document that is unavailable to those who enrol participants or assign interventions **see page 18-19 'Randomisation and blinding'**
Allocation concealment mechanism	16b	Mechanism of implementing the allocation sequence (eg, central telephone; sequentially numbered, opaque, sealed envelopes), describing any steps to conceal the sequence until interventions are assigned **n/a**
Implementation	16c	Who will generate the allocation sequence, who will enrol participants, and who will assign participants to interventions **see page 19 'Study procedures' (where applicable)**
Blinding (masking)	17a	Who will be blinded after assignment to interventions (eg, trial participants, care providers, outcome assessors, data analysts), and how **see page 18-19 'Randomisation and blinding'**
	17b	If blinded, circumstances under which unblinding is permissible, and procedure for revealing a participant's allocated intervention during the trial **n/a**

Methods: Data collection, management, and analysis

Data collection methods	18a	Plans for assessment and collection of outcome, baseline, and other trial data, including any related processes to promote data quality (eg, duplicate measurements, training of assessors) and a description of study instruments (eg, questionnaires, laboratory tests) along with their reliability and validity, if known. Reference to where data collection forms can be found, if not in the protocol **see page 19-22 'Study procedures' and 'Data collection'**
	18b	Plans to promote participant retention and complete follow-up, including list of any outcome data to be collected for participants who discontinue or deviate from intervention protocols **see page 21 (where applicable)**

For peer review only - http://bmjopen.bmj.com/site/about/guidelines.xhtml

Data management	19	Plans for data entry, coding, security, and storage, including any related processes to promote data quality (eg, double data entry; range checks for data values). Reference to where details of data management procedures can be found, if not in the protocol **see page 20 (where applicable)**
Statistical methods	20a	Statistical methods for analysing primary and secondary outcomes. Reference to where other details of the statistical analysis plan can be found, if not in the protocol **see page 22 'Statistical analysis'**
	20b	Methods for any additional analyses (eg, subgroup and adjusted analyses) **see page 22 'Statistical analysis'**
	20c	Definition of analysis population relating to protocol non-adherence (eg, as randomised analysis), and any statistical methods to handle missing data (eg, multiple imputation) **see page 22 'Statistical analysis'**
**Methods: Monitoring**		
Data monitoring	21a	Composition of data monitoring committee (DMC); summary of its role and reporting structure; statement of whether it is independent from the sponsor and competing interests; and reference to where further details about its charter can be found, if not in the protocol. Alternatively, an explanation of why a DMC is not needed **see page 22-23 'Safety'**
	21b	Description of any interim analyses and stopping guidelines, including who will have access to these interim results and make the final decision to terminate the trial **n/a**
Harms	22	Plans for collecting, assessing, reporting, and managing solicited and spontaneously reported adverse events and other unintended effects of trial interventions or trial conduct **see page 22-23 'Safety'**
Auditing	23	Frequency and procedures for auditing trial conduct, if any, and whether the process will be independent from investigators and the sponsor **see page 22-23 'Safety'**
**Ethics and dissemination**		
Research ethics approval	24	Plans for seeking research ethics committee/institutional review board (REC/IRB) approval **see page 23 'Ethics and dissemination'**
Protocol amendments	25	Plans for communicating important protocol modifications (eg, changes to eligibility criteria, outcomes, analyses) to relevant parties (eg, investigators, REC/IRBs, trial participants, trial registries, journals, regulators) **n/a**
Consent or assent	26a	Who will obtain informed consent or assent from potential trial participants or authorised surrogates, and how (see Item 32) **see page 19 'Study procedures'**

For peer review only - http://bmjopen.bmj.com/site/about/guidelines.xhtml

	26b	Additional consent provisions for collection and use of participant data and biological specimens in ancillary studies, if applicable **n/a**
Confidentiality	27	How personal information about potential and enrolled participants will be collected, shared, and maintained in order to protect confidentiality before, during, and after the trial **see page 20 'Data collection'**
Declaration of interests	28	Financial and other competing interests for principal investigators for the overall trial and each study site **see page 26 'Competing interests'**
Access to data	29	Statement of who will have access to the final trial dataset, and disclosure of contractual agreements that limit such access for investigators **see page 20 'Data collection'**
Ancillary and post-trial care	30	Provisions, if any, for ancillary and post-trial care, and for compensation to those who suffer harm from trial participation **See page 22-23 'Safety'**
Dissemination policy	31a	Plans for investigators and sponsor to communicate trial results to participants, healthcare professionals, the public, and other relevant groups (eg, via publication, reporting in results databases, or other data sharing arrangements), including any publication restrictions **see page 23 'Ethics and dissemination'**
	31b	Authorship eligibility guidelines and any intended use of professional writers **see page 23 'Ethics and dissemination'**
	31c	Plans, if any, for granting public access to the full protocol, participant-level dataset, and statistical code **see page 23 'Ethics and dissemination'**
**Appendices**		
Informed consent materials	32	Model consent form and other related documentation given to participants and authorised surrogates **Patient information form including model consent form are provided as Appendices for the editors.**
Biological specimens	33	Plans for collection, laboratory evaluation, and storage of biological specimens for genetic or molecular analysis in the current trial and for future use in ancillary studies, if applicable **n/a**

*It is strongly recommended that this checklist be read in conjunction with the SPIRIT 2013 Explanation & Elaboration for important clarification on the items. Amendments to the protocol should be tracked and dated. The SPIRIT checklist is copyrighted by the SPIRIT Group under the Creative Commons "Attribution-NonCommercial-NoDerivs 3.0 Unported" license.

For peer review only - http://bmjopen.bmj.com/site/about/guidelines.xhtml
